# Multi-Channel Metabolomics Analysis Identifies Novel Metabolite Biomarkers for the Early Detection of Fatty Liver Disease in Dairy Cows

**DOI:** 10.3390/cells11182883

**Published:** 2022-09-15

**Authors:** Xuan Zhang, Tingjun Liu, Xianpeng Hou, Chengzhang Hu, Letian Zhang, Shengxuan Wang, Qin Zhang, Kerong Shi

**Affiliations:** Key Laboratory of Animal Bioengineering and Disease Prevention of Shandong Province, College of Animal Science and Technology, Shandong Agricultural University, No. 61 Daizong Street, Taian 271018, China

**Keywords:** early diagnosis, metabolic disorder, metabolomics, biomarker, non-alcoholic fatty liver disease (NAFLD)

## Abstract

Fatty liver disease, a type of metabolic disorder, frequently occurs in dairy cows during the parturition period, causing a high culling rate and, therefore, considerable economic losses in the dairy industry owing to the lack of effective diagnostic methods. Here, metabolite biomarkers were identified and validated for the diagnosis of metabolic disorders. A total of 58 participant cows, including severe fatty liver disease and normal control groups, in the discovery set (liver biopsy tested, *n* = 18), test set (suspected, *n* = 20) and verification set (liver biopsy tested, *n* = 20), were strictly recruited and a sample collected for their feces, urine, and serum. Non-targeted GC-MS-based metabolomics methods were used to characterize the metabolite profiles and to screen in the discovery set. Eventually, ten novel biomarkers involved in bile acid, amino acid, and fatty acid were identified and validated in the test set. Each of them had a higher diagnostic ability than the traditional serum biochemical indicators, with an average area under the receiver operating characteristic curve of 0.830 ± 0.0439 (*n* = 10) versus 0.377 ± 0.182 (*n* = 9). Especially, combined biomarker panels via different metabolic pipelines had much better diagnostic sensitivity and specificity than every single biomarker, suggesting their powerful utilization potentiality for the early detection of fatty liver disease. Intriguingly, the serum biomarkers were confirmed perfectly in the verification set. Moreover, common biological pathways were found to be underlying the pathogenesis of fatty liver syndrome in cattle via different metabolic pipelines. These newly-discovered and non-invasive metabolic biomarkers are meaningful in reducing the high culling rate of cows and, therefore, benefit the sustainable development of the dairy industry.

## 1. Introduction

Fatty liver disease has a high incidence and has, for a long time, existed as a common type of metabolic disorder in periparturient and postpartum cows, which is caused by a negative energy balance, intense body fat mobilization and puerperal stress, thus seriously threatening the subsequent milk production and even the future reproductive capacity of dairy cows [1,2,3]. In the first month after calving, 5–10% of dairy cows had a severe form of fatty liver, and 30–40% had a mild or moderate fatty liver, and especially for the two weeks after calving, dairy cows carried the greatest risk for an outcome of a metabolic disorder [1,3,4]. It can be complicated by infectious diseases caused by immunodeficiency [5,6,7] and even other health and reproductive problems, causing a high culling rate of dairy cows in their perinatal period, and thus, considerable economic losses in the dairy industry of up to ten million dollars annually [8,9]. Ultrasonic imaging and/or traditional serum biochemical indicators (AST, GLU, INS, NEFA, etc.) were used in the production practice to distinguish cows with fatty liver disease; however, this was limited in application because of their low diagnostic sensitivity or high false-negative rates [10,11,12,13]. A liver biopsy continues to serve as the only reliable diagnostic method; it is not a practical method on farms, however, as it requires special training and leads to a high risk of infection [12,13]. Moreover, conducting a liver biopsy further aggravates the suffering of diseased cows. There is still a lack of effective diagnostic methods for fatty liver disease in dairy cattle with minimum invasions.

Circulating (serum or plasma) and terminal metabolite (milk, urine) biomarkers have been of recent great concern due to the identification of the molecular biomarkers facilitating the screening of metabolic disorders (such as ketosis, retained placenta, metritis, and lameness) in dairy cattle, and understanding the metabolism biology of cows during their parturition period [7,14,15,16,17,18,19,20]. However, few molecular markers have been identified for the occurrence of a fatty liver in dairy cows. Since fat deposition in the liver usually occurs ahead of other metabolic disorders, it is, therefore, urgent to discover novel biomarkers for the screening of fatty liver disease in cattle. We hypothesized that there is an inherent genetic regulation mechanism to regulate the occurrence of fatty liver disease in cattle. Our aims in this study were to develop novel non-invasive biomarkers (panel) to diagnose fatty liver diseased cows with greater specificity and sensitivity and to identify the common regulatory biological pathways that control the occurrence of fatty liver disease during the parturition period by detecting the terminal metabolite feces and urine, as well as the circulating metabolite (serum) using gas chromatography–mass spectrometry (GC–MS).

## 2. Materials and Methods

### 2.1. Study Design and Animal Participants

In the study, a total of 38 cows in their early postpartum period were enrolled and divided into the discovery and test sets. Each set included normal control cows (normal) and fatty liver disease cows (FL).

In the discovery set, the Holstein dairy cattle, being fed using the same management model throughout the experiment, were examined for the serum biochemical traits and thereby targeted for liver biopsies, according to the detailed procedures [7,19], so as to accurately diagnose the cows with fatty liver disease or a normal liver. The cows were diagnosed by the percentage of fat-deposited cells in the liver and then assigned to the normal or FL groups, with the result of less than 13% (*n* = 8) and more than 85% (*n* = 10), respectively. The percentage of fat-deposited cells was assessed via oil red staining of the biopsied liver tissues (Figure S1). It is worth mentioning that the values of the fatty-cell percentages (%) quantitated by the liver biopsy in the study were suggested to be positively correlated with liver TAG content (Figure S2). In contrast, the test cohort of cows (*n* = 20) was recruited from 178 postpartum candidates by identifying cows as having a high Pearson correlation coefficient of their own biochemical indicators with that of the biopsy-diagnosed cows. In other words, the cows possessing similar biochemical indicators with a fatty liver and/or healthy-liver cows in the discovery set were recruited into the test set. Information on the biochemical indicators of the enrolled cows is in Table S1.

**Figure 1:**
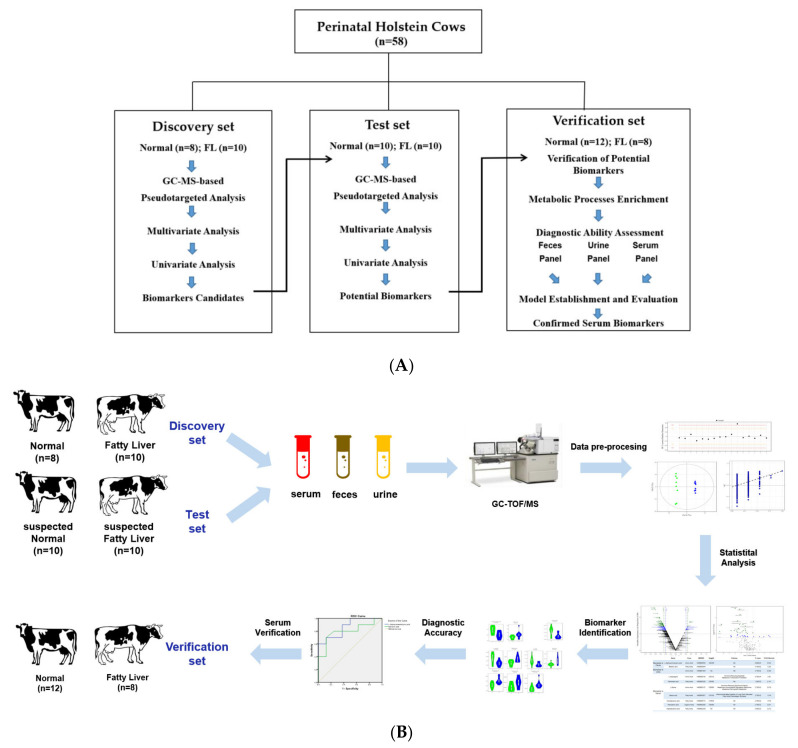
Design of the study. (**A**) Workflow and experimental design of the study. (**B**) Data analysis clue of the study.

The discovery set was first used to identify the candidate biomarkers via single- and multi-dimensional detection methods (Figure 1A). Then, the test set was used to further screen the biomarkers via the same method, finally defining the potential biomarkers, establishing a metabolite panel model, and evaluating its diagnostic performance and predictive ability (Figure 1B).

### 2.2. Serum, Urine, and Feces Sample Collection and Serum Biochemical Indicator Measurement

Serum, urine, and feces collection: Blood samples were collected from the tail vein of the hold dairy cows in the early morning before feeding at 7 ± 2 days after calving. The serum samples were then separated by centrifuging at 2500 rpm for 15 min after a 37 °C water bath incubation and immediately stored in liquid nitrogen for subsequent serum biochemical indicators detection. The urine and feces samples were collected using long-armed gloves and immediately put into liquid nitrogen for later use.

Serum biochemical indicator detection: The serum indices tested include: AST (aspartate aminotransferase), ALB (albumin), BHB (β-hydroxybutyric acid), GLU (glucose), INS (insulin), NEFA (non-esterified fatty acids), SUN (serum urea nitrogen), TCHO (total cholesterol), TG (triglyceride), TP (total protein), UA (urea acid), and UERA (urea nitrogen). The indicators were detected using reagent kits (Nanjing Jiancheng Biological Engineering Institute, Nanjing, China) using an automatic biochemical analyzer according to the detailed manufacturers’ procedures.

Liver tissue collection: The suspected biopsy cows were identified and selected according to their biochemical indicators, high NEFA, AST, and low GLU and INS levels. The biopsy operation was performed according to previous procedures [7,19]. The dairy cows were held in a cage and shaved on their side at the intersection of the 10th–11th rib and the middle humerus to the hip tubercle with an area of 5 × 5 cm. After sanitization and local anesthesia, the liver tissues were biopsied using the Bard Magnum biopsy system (Bard Peripheral Vascular, Inc., Tempe, AZ, USA), followed by surgical suturing of the skin. The animal was administered ketoprofen and penicillin G procaine by intravenous injection immediately after the biopsy and for the next 3 to 5 days until complete recovery of health. The liver tissue samples were immediately fixed in 5% polyformaldehyde for oil red O staining. Eventually, the biopsied dairy cattle were diagnosed with liver health conditions according to the average percentage of hepatic cells containing lipid droplets (Figure S1).

### 2.3. Sample Pre-Treatment and Non-Target GC–MS Analysis

The sample pre-treatment procedures referred to the previously published methods with minor modifications [21,22]. Briefly, each aliquot of the 50μL serum or urine sample was mixed with 10 μL of the internal standard and 175 μL of methanol/chloroform (*v*/*v* = 3:1). The sample was vortexed vigorously and centrifuged. Each 200 μL sample of supernatant was transferred to an autosampler vial and then evaporated using a vacuum concentrator. For the feces samples, each 5 mg of lyophilized feces sample was mixed with 10μL of the internal standard and extracted with 50μL of 50% methanol. The supernatant was carefully transferred to a microtube, and the residue was extracted again with 175 μL of methanol/chloroform (*v*/*v* = 3:1). The two-stepped supernatant was combined, and 100 μL of the mixed supernatant was transferred and evaporated. The evaporated samples were derivatized with 50 μL of methoxyamine (20 mg/mL in pyridine) at 30 °C for 2 h, followed by the addition of 50μL of MSTFA (1% TMCS) at 37.5 °C for another 1 h. Each 1μL of the derivatized sample was injected into a Pegasus HT GC–TOFMS system (Leco Corp., St. Joseph, MO, USA). Separation was performed on an Rxi-5 ms capillary column (30 m × 0.25 mm × 0.25 μm), and the oven temperature was set at 80 °C for 2 min, then ramped up to 300 °C at 12 °C/min, and maintained at 300 °C for 8 min. The mass spectra were collected with electron impact ionization (70 ev) at the full scan mode (*m*/*z* 50–500).

### 2.4. Quality Control Analysis and Model Reliability Testing

**Figure 2:**
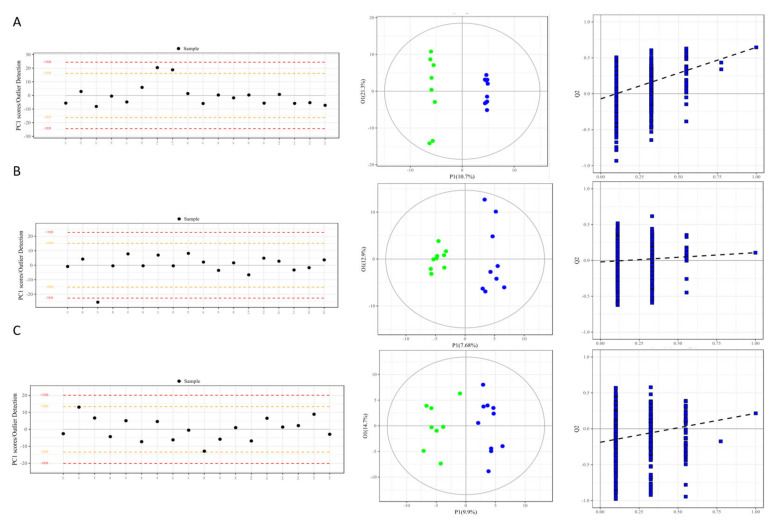
Quality control and model credibility assessment of samples in the discovery set (cows diagnosed by liver biopsy) from feces (**A**), urine (**B**) and serum (**C**). (**Left** panel) Multivariate control chart (MCC) shows the metabolite profiles of all individual feces (**A**), urine (**B**) and serum (**C**) samples in the discovery set. The black dot represents a single sample. The orange and/or red dashed line represents the biological quality control range limit. (**Middle** panel) Orthogonal partial least squares discriminant analysis (OPLS-DA) maps of the samples from feces (**A**), urine (**B**), and serum (**C**) reveal a significant separation of the fatty liver group from the normal control group, without any overlapping, indicating a successful model construction in the study. Green and blue dots represent normal and fatty liver samples, respectively. (**Right** panel) The predictive ability (Q2Y) of the model is shown in OPLS-DA maps of samples from feces (**A**, 0.998), urine (**B**, 0.952), and serum (**C**, 0.901). To avoid over-fitting of the model, 999 random permutation tests were performed so as to cross-validate the three components. Their intercept values were −0.071, −0.021, and −0.188 for feces (**A**), urine (**B**), and serum (**C**), respectively, thereby demonstrating the model’s effectiveness.

Multivariate quality control charts, an important means and tool for laboratory automation and quality management, were used in the study to testify to the credibility of our model because the charts are analyzed and established based on the scoring positions and the location tendency of all the samples (shown as Figure 2A). Further, a principal component analysis (PCA) and orthogonal partial least squares discriminant analysis (OPLS-DA) was also employed to test the reliability of the model and further distinguish the metabolic spectrum differences between the two groups in both the discovery set and test set (Figure 2B).

### 2.5. Data Analysis and Identification of Differentially Expressed Metabolites

The raw data generated by the GC–TOFMS were processed using a ChromaTOF (v4.71, Leco Corp., St. Joseph, MO, USA) for automated baseline denoising and smoothing, deconvolution, and peak alignment. Compound identification was performed by comparing both the MS similarity and FAMEs retention index distance with the referenced standards in the Jialib Mass library. The unsupervised principal component analysis model on the unit variance scale was used to evaluate the changes in the metabolic components and monitor the stability of the study. The supervisory model of the partial least squares discriminant analysis, based on one-dimensional variance scales, was employed to maximize the degree of dispersion between the disease and normal groups. According to its variable importance in projection (VIP) [23], the variables that significantly contributed to the classification were identified. Thereafter, 999 replacement tests [24,25] were conducted to assess the risk of over-fitting the model. A *t*-test [23] was used for the univariate analysis and a *p*-value < 0.05 indicated significance. The biomarker model was established and evaluated using the SPSS software, and the designed potential biomarker model was established and evaluated using binary logistic regression. A ROC (receiver operating characteristic) curve [26,27] was used to evaluate the results of the regression analysis, scoring the diagnostic specificity and sensitivity of the biomarker. The pathway analysis was performed based on a hypergeometric test [28] and KEGG (Kyoto encyclopedia of genes and genomes) pathway, displaying the significant biological differences between the disease and normal groups. A violin map test was used to verify the significant expression difference between potential biomarkers.

## 3. Results

### 3.1. Workflow of the Study and the Participants Cohort

The workflow and experiment designs of the study are shown in Figure 1A,B. To define the biomarker candidates, 18 cows, liver biopsied and with a detected fat deposition, were recruited into the discovery set. The fat deposition content was determined by Oil Red staining of the biopsied liver tissues. The diagnosed cows with a fat-deposited content of 13.17% ± 8.20% (*n* = 8) for the healthy controls and 84.71% ± 4.83% (*n* = 10) for the fatty liver disease group (Figure S1) were strictly selected, indicating their tremendous difference. To validate these biomarker candidates and define the potential biomarkers, a total of 20 cows, identified using their biochemical indicators by taking that possessed by biopsy-diagnosed fatty liver cows and/or healthy cows as the standard, were recruited into the test set. In other words, the cows possessing highly correlated biochemical indicators with a fatty liver and/or healthy-liver cows in the discovery set were harshly recruited into the test set, with Pearson correlation coefficients of 0.915 ± 0.041 in the suspected FL group (*n* = 10, *p* < 0.05) and 0.943 ± 0.092 in the suspected normal group (*n* = 10, *p* < 0.05), respectively. The biochemical indicators of the disease cows were significantly different to that of the normal cows, with elevated AST and NEFA (non-esterified fatty acids), and decreased GLU (glucose) levels. The detailed information is listed in Table S1.

### 3.2. Metabolic Profiling, Model Establishment and Evaluation

The results of the multivariate quality control charts showed that all the tested samples, including feces, urine, and serum, were located in an area of less than ×2 standard deviations along the *x*-axis, and the majority of the samples were close to the control limit (Figure 2A–C left). No abnormal values of these samples were undetected, indicating that the model in the study was reliable and the samples involved in the model were correct.

In order to further testify to the reliability of the data model and distinguish the difference in metabolic profiles between the disease and normal groups, principal component analysis and an advanced supervised discriminant model, orthogonal partial least squares discriminant analysis (OPLS-DA) were performed, and the results indicated that the disease group (blue dots) and the control group (green dots) in all feces, urine, and serum samples showed obviously separated aggregation community and significant differences (Figure 2A–C middle); the relatively high Q2Y values of the OPLS-DA analysis, evaluating the metabolites contributed to the group, indicating the credibility of the grouping models, with 0.918, 0.952 and 0.901 in the feces, urine, and serum samples, respectively. Further, to avoid the phenomenon of over-fitting caused by the supervised model, 1000 random sampling tests (Permutation test) on this model were performed to evaluate the reliability of the model (Figure 2A–C, right), and the Y-axis intercepts of less than zero in the three different samples confirmed the reliability of the represent study. The same situation was obtained by the samples in the test set (Figure S3). These variable metabolites, in both the discovery and test sets, were used for the subsequent multivariate and univariate analyses.

### 3.3. Identification of Candidate Metabolic Biomarkers in Feces, Urine, and Serum in the Discovery Set

A high-performance GC–MS analysis was used to detect the small molecular metabolites in the feces, urine, and serum of perinatal dairy cattle. The results showed that the measured functional metabolites were small organic molecules with similar classifications and proportions in every sample type, including amino acids and amines, organic acids, carbohydrates, fatty acids, and lipids, nucleosides, sugars, vitamins, and auxiliary factors (Figure S4). The obtained volume and category proportions of metabolites were, as expected in the project, supportive of the subsequent biomarker screening.

**Figure 3:**
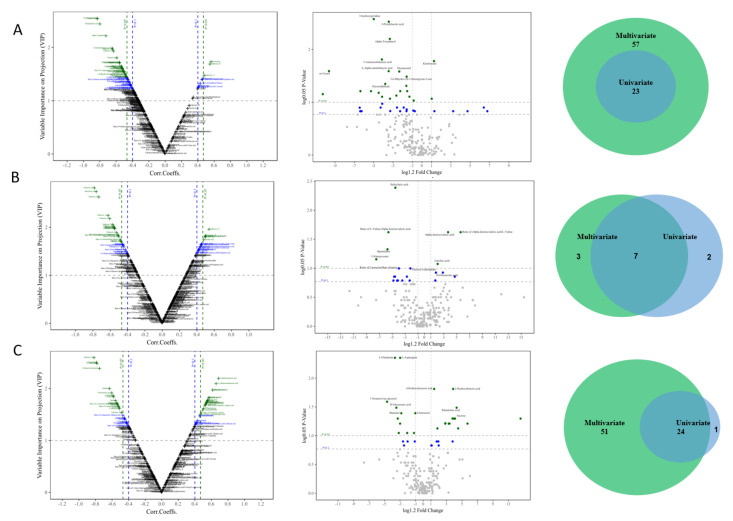
Identification of the differential expressing candidate metabolites in feces (**A**), urine (**B**) and serum (**C**) samples in the discovery set. (**Left** panel) The volcano plot (multi-dimensional analysis) revealed the differential expressing metabolites in feces (**A**), urine (**B**) and serum (**C**) samples through the multi-criteria assessment. The green and blue cross-shaped buddings represent the metabolites within the cutoff value of variable importance in the project (VIP) ≥ 1 and *p*-value < 0.05 and 0.01, respectively. The metabolites in the upper right corner of the image show upregulation in the fatty liver samples, and metabolites in the upper left corner show downregulation. (**Middle** panel) *t*-test map (single-dimensional analysis) of the fecal (**A**), urine (**B**), and serum (**C**) samples. The green dots represent the metabolites within the cutoff values of *p*-value < 0.05 and the log(fold change) ≥1.2. (**Right** panel) Venn diagram displays the differential expressing metabolites in the feces (**A**), urine (**B**), and serum (**C**) samples under multi-dimensional and single-dimensional data analysis. Green and blue represent the number of metabolites identified by single- and multi-dimensional analyses. More detailed information about the intersected common metabolites is listed in Tables S2–S4.

As described in Figure 1B, the first step of the screening process was to identify the candidate biomarkers from the discovery set. The candidate metabolite biomarkers with a VIP (variable importance in projection) of ≥ 1.0 and a *p* <0.05 on two principle components in each type of sample were firstly identified via a V-plot volcanic map (Figure 2A–C, left). Subsequently, a univariate analysis was used to determine whether these metabolites were significantly altered via the single dimension statistical method *t*-test, with an FC (fold change) of > 1.2 and a *p* < 0.05 in the disease groups compared with the normal control groups (Figure 2A–C, middle). Finally, 23 candidate biomarkers in feces (Figure 3A, right), 7 in urine (Figure 3B, right), and 24 in serum (Figure 3C, right) were identified as important variables that contributed to the classifications in the discovery set, and mainly included amino acids, fatty acids, carbohydrates, and organic acid. The detailed information on these differential metabolites is listed in Tables S2–S4.

### 3.4. Defining Potential Metabolic Biomarkers for Fatty Liver Disease

An independent test cohort of 20 cows (Figure 1), named the test set, was used to evaluate the reliability of these biomarker candidates and define the useful biomarkers. First, the same analysis methods and procedures were applied as per that in the discovery set (Figure S5A,B). Thus, 24 candidate biomarkers in feces (Figure S5A, right), 10 in urine (Figure S5B, right), and 26 in serum (Figure S5C, right) were identified as important variables that contributed to the classifications in the test set. The detailed information on these differential metabolites is listed in Tables S5–S7.

Second, the common candidate biomarkers, as potential biomarkers, were separately defined via intersecting the discovery set and test set from the feces, urine, and serum samples, respectively. The defined potential metabolite biomarkers must satisfy the following criteria: (1) significant differences between the normal and FL groups; (2) simultaneously consistent change direction between the normal and FL groups. Ultimately, two metabolites were retained in the feces: L-alpha-aminobutyric acid and behenic acid; one metabolite was retained in urine: 3-nitrotyrosine; and seven metabolites were retained in the serum: L-asparagine, palmitoleic acid, L-serine, stearic acid, nonadecanoic acid, petroselinic acid, and heptadecanoic acid. Their detailed information is shown in Table 1.

**Figure 4:**
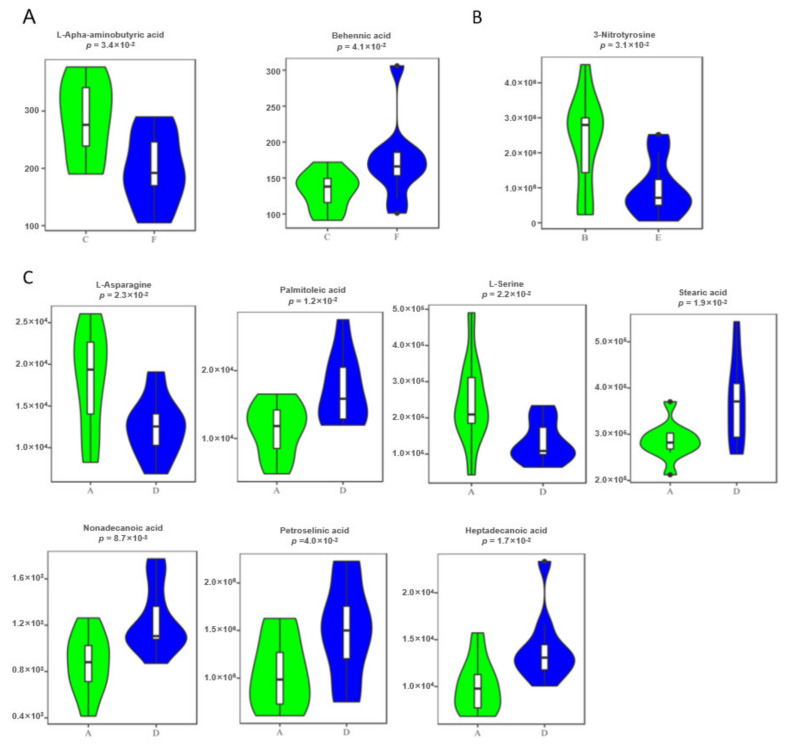
Violin charts showing the identified potential diagnostic biomarkers having significantly different expression levels between fatty liver cows and normal controls in both the discovery set and test set. (**A**) Two feces markers, L-alpha-aminobutyric acid (left) and behenic acid (right); letters “C” and “F” represent control and disease feces, respectively. (**B**) One urine marker, 3-nitrotyrosine; letters “B” and “E” represent control and disease urine, respectively. (**C**) Seven serum markers, L-asparagine, L-serine, palmitoleic acid, and stearic acid (upper panel, left to right); nonadecanoic acid, petroselinic acid, and heptadecanoic acid (lower panel, from left to right). Letters “A” and “D” represent control and disease serum, respectively.

These metabolites were all shown to have significantly different expressions in the FL groups from the normal control groups (Figure 4), suggesting the ideal biomarkers to distinguish fatty liver disease cows from healthy control subjects. The fecal marker L-alpha-aminobutyric acid was significantly decreased in the FL group compared to the control group (fold change 0.534, *p* = 0.0085), while behenic acid was significantly increased (fold change 1.203, *p* = 0.041). The urine marker, 3-nitrotyrosine, was significantly decreased in the disease group. Two serum markers, L-asparagine and L-serine, had significantly lower expression levels in the disease group, while the other five had significantly higher expression levels in the disease group; they were palmitoleic acid, stearic acid, nonadecanoic acid, petroselinic acid, and heptadecanoic acid (Table 1). These increased NEFAs were also observed in early postpartum dairy cows.

### 3.5. Validation of the Metabolic Marker Panel in the Test Set

**Figure 5:**
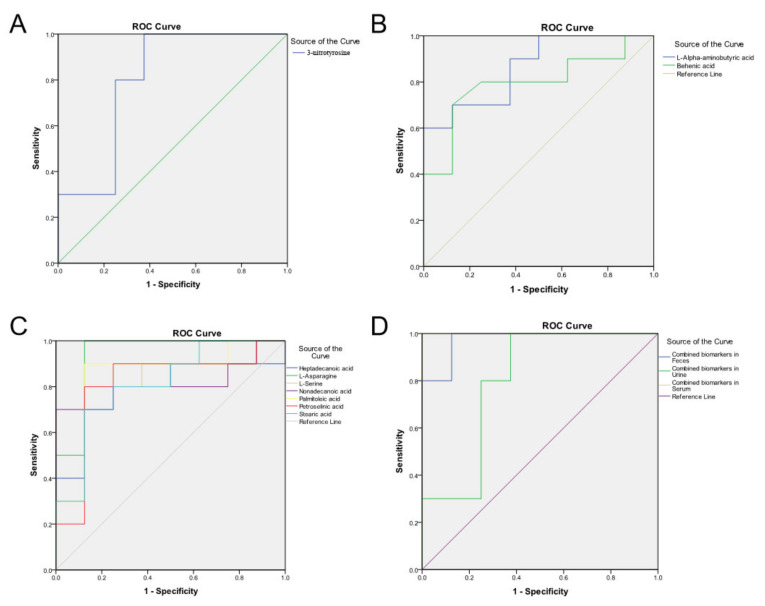
Validation of diagnostic performance of the identified ten biomarkers for metabolic disorders using ROC (receiver operating characteristic) curve (permutation test *p-*values < 0.05). (**A**) ROC curve of urine biomarker, 3-nitrotyrosine. (**B**) ROC curve of the two feces biomarkers, L-alpha-aminobutyric acid (blue line) and behenic acid (green line); (**C**) ROC curve of the seven feces biomarkers, heptadecanoic acid (blue line), L-asparagine (green line), L-serine (grey line), nonadecanoic acid (purple line), palmitoleic acid (yellow line), petroselinic acid (red line), and stearic acid (light blue line). (**D**) ROC curve of the combined biomarker panel in feces (blue line), urine (green line), and serum (grey line). The line with a 45° slope in each panel is the reference boundary line with an AUC (area under the curve) of 0.5, meaning the diagnostic method is effective or not. The higher the AUC value is, the higher the accuracy of the diagnostic method. The AUC values are listed in Table 2.

In order to verify the diagnostic performance of this metabolite panel for fatty liver disease in dairy cattle, an ROC (receiver operating characteristic) curve was established. The results indicated that these metabolites in feces, urine and/or serum were all showing a higher sensitivity and similar specificity to identify the FL cows from the normal control cows (Figure 5, permutation test *p*-values < 0.05). For example, fecal metabolite L-alpha-aminobutyric acid, behenic acid, and urine metabolite 3-nitrotyrosine obtained AUC values of 0.863, 0.794, and 0.802, respectively (Table 2, Figure 5A,B). When combining and forming a metabolite panel for each type of sample, it showed better diagnostic performance, with higher AUC values than any single metabolite, such as 0.975 of the combined two biomarkers in feces and 1.000 of the combined seven biomarkers in serum (Table 2, Figure 5D). Especially, a fairly outstanding AUC value of 0.988 was obtained if combining the three metabolite biomarkers in both feces and urine, which are considered non-invasive diagnostic biomarkers (Table 2, Figure 5D). Meanwhile, the diagnostic performance of these potential biomarkers was notably higher (AUC of 0.830 ± 0.0439, *n* = 10) than that of the traditional serum biochemical indicators (0.377 ± 0.182, *n* = 9) (Table 2, Figure S6). Especially, even though AST was the most classic serum index to indicate a fatty liver (AUC = 0.756, Table 2, Figure S6), every single novel identified biomarker was still better than AST. The strict stepwise screening and validation thus identified reliable metabolite biomarkers for the early diagnosis of cows with fatty liver disease.

### 3.6. Associated Biological Pathways of Metabolite Biomarkers with Fatty Liver Syndrome in Cattle

**Figure 6:**
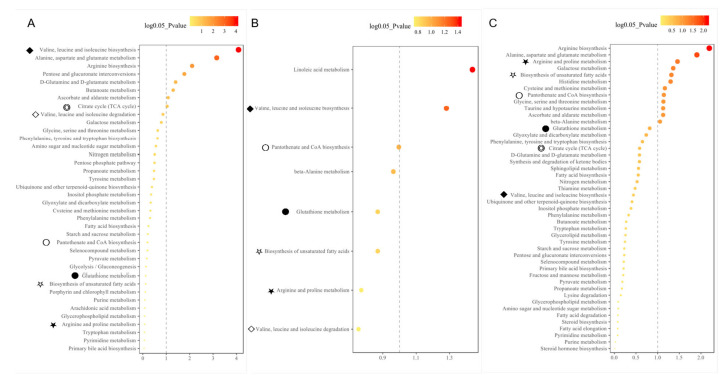
Common biological metabolic pathways were enriched by differential expressing metabolites identified from feces (**A**), urine (**B**) and serum (**C**). The common pathways enriched by different metabolites identified from feces, urine and/or serum were indicated using different symbols (◆, ◇, ★, ☆, ○, ◎ and/or ●). Red represents the significance of the pathway when it was enriched; the redder it is, the more significant (with a smaller *p*-value) the pathway is. Dot size represents the number of metabolites involved in the pathway; the bigger the dot is, the more metabolites are involved.

To understand the metabolite-related pathways underlying the pathogenesis of metabolic disorders in dairy cattle, metabolic pathway enrichment analyses (MPEA) of all candidate differential metabolites were carried out in both the discovery and test sets. The results showed that the metabolic disorders in postpartum dairy cows with fatty livers were mainly related to as follows: arginine biosynthesis; alanine, aspartate, and glutamate metabolism; arginine and proline metabolism; biosynthesis of unsaturated fatty acids; histidine metabolism; cysteine and methionine metabolism; pantothenate and CoA biosynthesis; glycine, serine, and threonine metabolism; taurine and hypotaurine metabolism; beta-alanine metabolism; and pentose and glucuronate interconversion in feces (Table S8). The above pathways are mainly related to the metabolism of bile acids, amino acids, and lipids during the progression of metabolic disorders. Meanwhile, these enriched metabolic pathways revealed the effects of energy metabolism, oxidative stress, and inflammation on the pathogenesis of metabolic disorders in dairy cows, especially fatty liver disease. Intriguingly, several common metabolic pathways were simultaneously enriched from feces, urine, and circulating serum, and three different types of metabolic systems: valine, leucine, and isoleucine biosynthesis; pantothenate and CoA biosynthesis; glutathione metabolism; biosynthesis of unsaturated fatty acids; arginine and proline metabolism (Figure 6). This implicates the common metabolism pathways underlying the pathogenesis of metabolic disorders in dairy cattle, especially fatty liver syndrome.

### 3.7. Verification of Serum Biomarkers in a Third Liver Biopsy-Diagnosed Holstein Population

**Figure 7:**
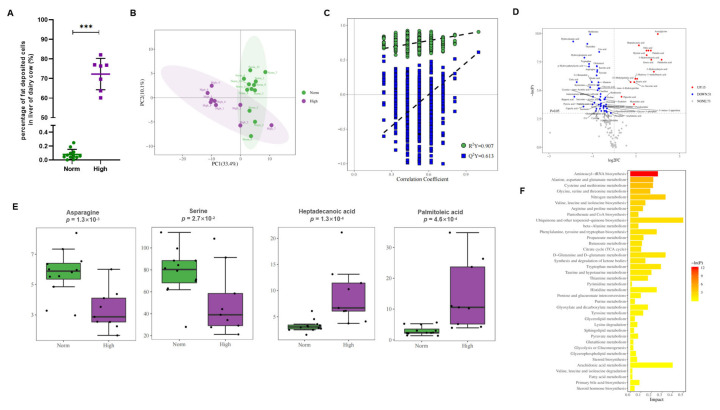
Verification of the serum diagnostic marker in a third biopsied population. (**A**) New serum samples from a third dairy cow population in their early postpartum period; a total of 20 individuals were diagnosed for their liver health by liver biopsy, followed by oil red staining. The normal group (Norm, *n* = 12) and fatty liver group (High, *n* = 8)) were diagnosed with 0.080% ± 0.073% and 72.25% ± 7.99% of cells deposited with fat, respectively. (**B**) PCA analysis results of the 20 serum samples reflect reduced levels of variation in the data set. (**C**) OPLSDA modeling validation results of the new samples, with R2Y and Q2Y of 0.907 and 0.613, respectively. (**D**) Volcano plot of univariate analysis, identifying differential expressing metabolites in serum samples, including 5 up-regulated (in red dots) and 51 down-regulated (in blue dots). (**E**) Boxplot of representative up-regulated (Heptadecanoic acid and Palmitoleic acid) and down-regulated (Asparagine and Serine) metabolites in the fatty liver group confirmed their diagnostic sensitivity and specificity in fatty liver disease. (**F**) Similar biological metabolic pathways were enriched by differential expressing metabolites identified in the third population, such as arginine and proline metabolism, TCA cycle, valine, leucine, and isoleucine biosynthesis. *** *p* < 0.001.

A third Holstein population, a total of 20 dairy cattle in their early postpartum period, were diagnosed for their liver health by liver biopsy and subsequent oil red staining. The normal group (Norm, *n* = 12) and fatty liver group (High, *n* = 8)) were diagnosed with 0.080% ± 0.073% and 72.25% ± 7.99% of cells deposited with fat, respectively (Figure 7A). Non-targeted metabolomics was again used to identify the differentially expressed metabolites in serum by the GC-TOFMS method. The PCA and OPLS-DA modeling validation analyses demonstrated the model’s effectiveness (Figure 7B,C). The differential expressing metabolites were finally obtained by intersecting the metabolite set identified by OPLSDA-VIP and univariate statistics analyses (Figure 7D and Figure S7), following the threshold value for the differential metabolites selection: *p* < 0.05 and |log2FC| >= 0. The identified novel biomarkers, such as the fatty acids, Heptadecanoic acid and Palmitoleic acid, and amino acids, L-Asparagine and L-Serine, were perfectly verified to be up- and down-regulated in the fatty liver group (Figure 7E), respectively, confirming their diagnostic sensitivity and specificity in fatty liver disease in dairy cattle. In addition, the common biological metabolic pathways were again enriched by the differential expressing metabolites identified in the third population, such as arginine and proline metabolism, TCA cycle, valine, leucine, and isoleucine biosynthesis (Figure 7F).

## 4. Discussion

Metabolic disorders of dairy cattle, such as fatty liver disease and ketosis, in the transition period, remain as prevalent now as they did 20 years ago [5]. Nowadays, metabolomics approaches are boomingly applied for the identification of metabolite panels in circulating and/or terminal metabolites in dairy cows that can differentiate metabolic disorder cows from healthy cows, including ketosis, retained placenta, metritis, lameness, mastitis, or displaced abomasums [9,14,15,16,17,18,19,20,29]. This study used non-targeted metabolomics and identified several predictive and diagnostic biomarker panels of fatty liver disease. The associated metabolic pathways possibly involved during the onset and progression of fatty liver disease are also discussed below.

### 4.1. Desirable and Novel Metabolite Biomarkers (Panels) to Early Diagnose Fatty Liver Cattle Were Strictly Identified in the Study

Metabolomics is defined as a comprehensive and fully quantitative analysis of all detectable metabolites (especially for small molecular weight molecules) within a particular biological sample to indicate an overview of metabolic status, which can provide new insight into the pathological mechanisms in diseases. Nuclear magnetic resonance (NMR) spectroscopy and MS techniques are the most commonly applied analytical platforms for metabolomics studies [17,18,29,30]. In comparison with NMR spectroscopy, the major advantage of MS is the higher selectivity and sensitivity, as MS can measure analytics routinely in the femtomolar to the attomolar range. In MS-based metabolomics, GC–MS is the most frequently used platform because it offers structural information, high throughput, reasonable quantitative precision, relatively high reproducibility, and resolution (the sensitivity is at least two orders of magnitude higher than NMR). In the present study, the GC-TOFMS-based metabolomics of body fluids (serum and urine) and terminal metabolites (feces) were processed. Novel and desirable metabolite biomarkers (panels) for the screening and/or early diagnostic of fatty liver cattle were strictly identified in the study and validated as having a higher accuracy (specificity and sensitivity). Firstly, liver biopsy-diagnosed dairy cows, named as the discovery set, were applied for the primary identification of metabolite biomarker candidates (Figure 1, Figure 2 and Figure S1). Secondly, the biomarker candidates were further testified (screened) by the highly suspected dairy cows in the test set (Figure 1 and Figure S3), who had consistent serum biochemical indicators with the biopsied diagnosed cows in the discovery set. Thirdly, after systematic, rigorous selection using multivariate and univariate statistical analyses (Figure 3 and Figure S5), the identified potential biomarkers were confirmed to be significantly differentially expressed in the disease and normal animals by the violin map test (Figure 4). Finally, ROC curves were established to validate the diagnosis performance of these potential biomarkers (Figure 5 and Figure S6). Among the 10 identified biomarkers, every single one had a higher diagnostic performance than the traditional serum indicators (0.830 ±0.0439, *n* = 10 versus 0.377 ± 0.182, *n* = 9; Table 2). Furthermore, the combined biomarkers had an even higher diagnostic sensitivity and specificity (AUC 0.975, 0.988, and 1.000, Table 2) for fatty liver dairy cows. It is important that the identified biomarkers, such as Heptadecanoic acid and L-Serine (Figure 7), were perfectly verified in a third biopsy-diagnosed population by non-targeted metabolomics, with 12 samples from healthy liver individuals and 8 from fatty liver cows. Moreover, common biological metabolic pathways were enriched by the differential expressing metabolites identified in the third population, such as arginine and proline metabolism, TCA cycle, valine, leucine, and isoleucine biosynthesis, again confirming the reliability of the novel identified biomarkers. In sum, the resounding successful experiment design, refined analysis methods, and rigorous validation highlight the meaningful reliability of the novel biomarkers identified in this study. Specifically, other than the advantage of higher diagnostic sensitivity and specificity of these identified biomarkers, non-invasiveness, rapidity, and convenience would be advocated in consideration of the issue of animal welfare and the concept of a healthy and sustainable dairy industry. This is because the diagnosis biomarkers can be detected by using terminal metabolites, feces, and urine. The identified non-invasive biomarkers would offer more potential and power to be utilized in production practices.

### 4.2. Dysregulated Fatty Acid Metabolism and Impaired Metabolism Capacity Were Accompanied with Fatty Liver Cattle

Increased fatty acid levels and decreased amino acid levels were shown in fatty liver animals compared to healthy ones based on different metabolic systems, no matter the exosomatic metabolite feces and urine or circulating serum. Specifically, among the ten identified novel biomarkers, four of them are amino acids, which showed significantly suppressed expression levels in the FL disease group; the other six are fatty acids, which showed significantly enhanced expression levels in the FL disease group (Table 1), which was verified in a third biopsied Holstein population (*n* = 20, Figure 7). The phenomenon of increased non-esterified fatty acids (NEFAs) has been shown to be closely related to the pathology of fatty liver disease in the perinatal period of dairy cattle, which usually results from a negative energy balance [31,32]. In the postpartum period, the lactation of the mammary gland slowly became increased and therefore increased the body lactose consumption, easily causing the cow to experience an insufficient sugar supply, thus promoting body fat mobilization. However, enhanced fat mobilization causes a dramatic increase in NEFA in the liver and circulating serum [4,33]. Excessive NEFAs in circulating serum that cannot be oxidized will be re-esterified to synthesize triglycerides (TG) in the liver, which are difficult to transport out of the liver because of the lower activity of esterase in cattle, leading to an excessive accumulation of TG in the liver [1,34,35,36]. Therefore, the increased circulating fatty acids could indicate the occurrence of fatty liver syndrome [17,19,35]. For example, serum palmitic acid was shown to increase in fatty liver disease cows (Table 1). The study showed that the decrease in palmitic acid oxidation in the liver tissue positively correlated with the increase in triglycerides [37] and negatively correlated with Decanoyl, which inhibits β-oxidation, blocking the ketogenesis of severe ketonemia, another type of metabolic disorder in dairy cows [37]. On the other hand, the decreased amino acid level was also evidenced to be associated with metabolic disorders. For example, the amino acid marker L-α-aminobutyric acid in feces, mainly involved in the metabolism of cysteine and methionine (Figure 6A,C and Figure 7), was significantly reduced in the diseased group, indicating increased steatosis. Previous evidence showed that mice with a methionine metabolism-related gene deficiency developed liver diseases, mainly fatty livers and liver cancer [38,39]. Another amino acid biomarker in urine was also confirmed to be associated with liver injury. For example, nitrotyrosine is a specific marker generated by endogenous peroxynitrite anion (ONOO-), exhibiting different expressions in different body fluids and species. ONOO in the serum, as a strong oxidant, can effectively oxidize the tyrosine residues at the thiol and iron/sulfur (Fe/S) center, thereby inactivating the target proteins or enzymes, inhibiting respiratory enzymes, and destroying the mitochondrial structure, thus significantly inhibiting the ability of the antioxidant enzymes to scavenge oxygen-free radicals, initiating lipid peroxidation, and participating in liver metabolic disorders and disease [40]. Accordingly, an enhanced fatty acid metabolism and suppressed uptake capacity would be two important pathological features of fatty liver cattle.

### 4.3. Common Biological Pathways Were Underlying the Pathogenesis of Fatty Liver Syndrome in Cattle

The biological pathway enrichment analysis revealed that the metabolic reprogramming was mainly related to the metabolism of fatty acids, amino acids, and bile acids during the pathogenesis of fatty liver disease in dairy cattle (Tables S2–S8), such as the biosynthesis of unsaturated fatty acids, primary bile acid biosynthesis, valine, leucine, and isoleucine degradation/biosynthesis (Figure 6). These common pathways, enriched by different resourced metabolites, provide insights into the underlying pathogenesis of fatty liver disease, such as the perturbations of energy metabolism, oxidative stress, and inflammation. In this study, the amino acid metabolisms/biosynthesis pathways were concurrently enriched by the differential expressed metabolites from feces, urine, and/or serum, such as glutathione metabolism; valine, leucine, and isoleucine biosynthesis/degradation; arginine and proline metabolism; etc (Figure 6 and Figure 7, Table S8). Amino acid metabolism pathways have been confirmed to be associated with the development of ketosis and fatty liver disease by previous studies [17,41]. Firstly, these amino acid metabolisms were closely associated with glucogenesis and/or adipogenesis via the TCA cycle (tricarboxylic acid cycle) by synthesizing Acetyl CoA, α-ketoglutaric acid, succinyl CoA, fumaric acid, and/or oxaloacetic acid. The dysregulation of the amino acid metabolism would cause abnormal levels of ketone body—AST/ALT in the body fluids, which usually implicates liver injury. Secondly, special amino acids were shown to be associated with liver disease. For example, arginine, as a conditionally essential amino acid, has a certain regulatory effect on lipid metabolism, such as white/brown fat mass. Especially, L-arginine ethyl ester and chenodeoxycholic acid conjugate (cdcarg) were developed into novel bile acid molecules to treat liver disease, non-alcoholic fatty liver disease (NAFLD), and non-alcoholic steatohepatitis (NASH) [42,43]. Additionally, bile acids play important roles in energy metabolism, oxidative stress, and inflammation, being significantly associated with the levels of AST, ALT, and bilirubin [23,44]. Asparagine could prevent liver triglyceride elevation. A long-term diet deficient in choline and methionine could cause hepatocellular carcinoma and other liver diseases [38,39,45]. In the present study, various amino acid metabolism pathways were significantly enriched, such as glycine, serine, threonine metabolism, alanine, aspartate, and glutamate metabolism, which are involved in glutathione metabolism. Interestingly, the glutathione metabolism was simultaneously found to be dysregulated in body fluids (serum, urine) and feces (this study, Figure 6) and also liver tissues (our previous studies) [7,19]. Studies have shown that the deletion of the GNMT (glycine N-methyltransferase) gene leads to the development of a fatty liver and fibrosis in mice [46,47]. GNMT was found to be silent in human liver cancer and down-regulated in the liver of patients at risk of liver cancer, such as hepatitis C virus and alcohol-induced cirrhosis [38,48], suggesting its key role in maintaining liver health [49,50]. Therefore, the evidence that cows with fatty liver disease experienced fluctuations of metabolism involved in amino acids, fatty acids metabolism, oxidative stress and inflammation [7,17,41] would be supportive of the complement of a full picture of energy metabolism in transition dairy cattle, so as to reveal the etiopathology of the disease.

### 4.4. The Identified Serum Biomarkers Were Confirmed by a Third Biopsied Population

The metabolomics in this study provides a practical strategy for screening fatty liver disease cows in their transition period using a small amount of feces, urine, or serum. The screening of fatty liver disease may be used as an index for the routine examinations of cows on commercial farms. In this study, the identified serum biomarkers, such as Heptadecanoic acid and L-Serine (Figure 7), were perfectly verified in a third biopsy-diagnosed population by non-targeted metabolomics, with 12 samples taken from healthy liver individuals and 8 samples from fatty liver cows. Moreover, common biological metabolic pathways were enriched by the differential expressing metabolites identified in the third population, such as arginine and proline metabolism, TCA cycle, valine, leucine, and isoleucine biosynthesis, again confirming the reliability of the novel identified biomarkers.

Although the reliability of the novel identified biomarkers in the study was verified, the novel biomarkers identified from the feces, urine, and serum in this study might be single-faceted or limited due to the trade-off between coverage, throughput, and cost. To gain a relatively wide metabolite coverage, a more effective and robust mass spectrum-based metabolomics method could be attempted in the future. In addition, although a higher diagnostic sensitivity and specificity of these identified biomarkers were obtained in all the studied populations of Holstein, their application in other dairy breeds needs to be further investigated.

### 4.5. Potentiality of the Study

When non-alcoholic fatty liver disease (NAFLD) occurs in human beings, it is usually accompanied by metabolic disorder syndromes and obesity, similar to fatty liver cows having increased plasma insulin and fatty acid concentration, elevated fasting aminotransferase (aspartate aminotransferase, AST/alanine aminotransferase, ALT; Table S1) and/or triglycerides (TG) level, and also an abnormal lipid accumulation in the liver (Figure S1) [51,52,53]. Even though the different origins of fatty acids caused by altered lipid homeostasis (lipolysis) and different insulin resistance status would differ NAFLD in patients from fatty liver disease in cattle, in view of the basal metabolism pathways, dairy cows with fatty liver disease, as an animal model, could bring inspiration on the etiopathology of typical NAFLD. Nowadays, NAFLD and its complications are currently recognized as a major health threat worldwide [54,55]. Recently, metabonomics involving the study of the entire metabolome [56] has been identified as a promising and powerful tool for detecting disease progression, elucidating its pathology, and assessing the impact of drugs on certain pathological conditions [23,30,57]. Notably, the application of metabolomic technology in screening for the biorecognition markers in fatty liver disease has been studied initially, and most of them have appeared in NAFLD research. For example, the combination of betaine and propionyl carnitine in the serum can make for a good prediction of liver diseases and can be used as a supplementary diagnosis method for the clinical fatty liver cancer diagnostic marker, alpha-fetoprotein (AFP) [58]. Through a multicenter cross-sectional study, a biomarker combination consisting of Phe-Trp and GCA was identified and used as a test for hepatocellular carcinoma (HCC), and they were further developed as an effective tool to verify AFP false-negative HCC patients and high-risk S-HCC patients [23]. The identification of novel and non-invasive diagnosis biomarkers for the metabolic disorders in the study, especially for fatty liver diseased dairy cattle, would be inspired and supportive for revealing the pathology and pathogenesis of NAFLD [53,54].

## 5. Conclusions

In summary, novel biomarker panels consisting of amino acids and fatty acids were defined and validated as an effective tool for detecting metabolic disorders (fatty liver disease) in dairy cows by non-targeted GC–MS metabolomics determination. The biomarkers could discriminate the diseased cows with a much higher diagnostic sensitivity and specificity than that of the traditional serum biochemical indicators. Moreover, the non-invasive markers will have utilization potential in terms of animal welfare issues, which are nowadays highly advocated internationally. Therefore, we believe these metabolic biomarkers are meaningful for a reduction in high culling rates of cows during their early lactation period and also for the healthy and sustainable development of the dairy industry.

## Figures and Tables

**Table 1:** Basic information of the identified potential biomarkers that are significantly differential, expressing in the fatty liver group and normal group of both the discovery set ^1^ and test set ^2^ in feces, urine, and/or serum.

Biomarker Source	Biomarker Name	Class	FC (FL/Norm) ^3^	*p*-Value	HMDB ID ^4^	KEGG ID ^5^	Associated Pathways
Feces	L-Alpha-aminobutyric acid	Amino Acid	0.534	8.50 × 10^−3^	HMDB00452	C02356	/
	Behenic acid	Fatty Acids	1.203	4.10 × 10^−2^	HMDB00944		/
Urine	3-Nitrotyrosine	Amino Acid	0.256	3.10 × 10^−2^	HMDB01904	/	/
Serum	L-Asparagine	Amino Acid	0.58	8.70 × 10^−4^	HMDB00168	C00152	Ammonia Recycling; Aspartate Metabolism; Transcription/Translation
	Palmitoleic acid	Fatty Acids	2.191	1.20 × 10^−2^	HMDB03229	C08362	/
	L-Serine	Amino Acid	0.579	2.70 × 10^−2^	HMDB00187	C00065	Ammonia Recycling; Glycine and Serine Metabolism; Homocysteine Degradation; Methionine Metabolism; Sphingolipid Metabolism
	Stearic acid	Fatty Acids	1.819	2.70 × 10^−2^	HMDB00827	C01530	Mitochondrial Beta-Oxidation of Long Chain Saturated Fatty Acids; Plasmalogen Synthesis
	Nonadecanoic acid	Fatty Acids	1.678	2.70 × 10^−2^	HMDB00772	C16535	/
	Petroselinic acid	Organic Acids	2.831	2.70 × 10^−2^	HMDB02080	C08363	/
	Heptadecanoic acid	Fatty Acids	2.272	3.40 × 10^−2^	HMDB02259	/	/

^1^ Discovery set, samples from cows diagnosed by liver biopsy. ^2^ Test set, samples from cows diagnosed by serological detection.^3^ FC, fold change, the relative expression level of the metabolite in the fatty liver group (FL) compared to that in the normal group (Norm).^4^ HMDB ID: ID number of the metabolite in the Human Metabolome Database. ^5^ KEGG ID: ID number of the metabolic pathway in the biological information database.

**Table 2:** Diagnostic performance assessment of the identified metabolic biomarkers and the traditional biochemical indicators using the ROC (receiver operating characteristic) curve.

Biomarker Source	Biomarker Name	AUC in Discovery Set (95% CI) ^2^	AUC in Test Set (95% CI) ^3^	*p*-Value in Violin Chart ^4^
Traditional Biochemical Indicator in Serum ^1^	AST	0.756	/	/
	UREA	0.5	/	/
	ALB	0.469	/	/
	INS	0.363	/	/
	UA	0.338	/	/
	TP	0.313	/	/
	TG	0.294	/	/
	TCHO	0.25	/	/
	GLU	0.112	/	/
Biomarker in Feces	L-Alpha-aminobutyric acid	0.863	0.825	3.40 × 10^−2^
	Behenic acid	0.794	0.929	4.10 × 10^−2^
Combined biomarkers in Feces	/	0.975	1	/
Biomarker in Urine	3-Nitrotyrosine	0.802	0.841	3.10 × 10^−2^
Combined biomarkers in Feces and Urine	/	0.988	1	/
Biomarker in Serum	L-Asparagine	0.938	0.76	2.30 × 10^−2^
	Palmitoleic acid	0.85	0.81	1.20 × 10^−2^
	L-Serine	0.812	0.79	2.20 × 10^−2^
	Stearic acid	0.813	0.79	1.90 × 10^−2^
	Nonadecanoic acid	0.813	0.84	8.70 × 10^−3^
	Petroselinic acid	0.813	0.76	4.00 × 10^−2^
	Heptadecanoic acid	0.8	0.84	1.70 × 10^−2^
Combined Biomarkers in Serum	/	1	1	/

^1^ AST, aspartate aminotransferase; UREA, urea nitrogen; ALB, albumin; INS, insulin; UA, urea acid; TP, total protein; TG, triglyceride; TCHO, total cholesterol; GLU, glucose. ^2^ AUC (area under the curve) values calculated from the biomarkers from the discovery set (samples from cows diagnosed by liver biopsy) upon ROC (receiver operating characteristic) curve construction with 95% CI (confidence interval). ^3^ AUC values calculated from biomarkers from the test set (samples from cows diagnosed by serology), with 95% CI (confidence interval). ^4^ *p*-value from the Violin Chart, indicating the biomarker has a significant statistical difference in expression levels between fatty liver and normal cows.

## Data Availability

The data used to support the findings of this study are available from the corresponding author upon request.

## Supplementary Materials

The following supporting information can be downloaded at: https://www.mdpi.com/article/10.3390/cells11182883/s1, Figure S1: Oil red O staining revealed the significant lipid accumulation in the fatty liver tissue (C,D), compared with normal control animal (A,B). Red represents stained fat in the cell; Figure S2: The TAG content (nmol/g protein) in liver were assessed in representative normal of fatty liver samples, indicating that the values of fatty cell percentage (%) quantitated by liver biopsy in the study were positively correlated with liver TAG content; Figure S3: Quality control and model credibility assessment of samples in the Test set (cows strictly selected by serological detection) from feces (A), urine (B) and serum (C). (Left panel) Multivariate control chart (MCC) shown the metabolite profiles of all individual feces (A), urine (B) and serum (C) samples in the Discovery set. Black dot represents a single sample. Orange and/or red dashed line represents biological quality control range limit. (Middle panel) Orthogonal partial least squares discriminant analysis (OPLS-DA) maps of the samples from feces (A), urine (B) and serum (C), revealing a significant separation of fatty liver group from normal control group, without any overlapping, indicating a successful model construction in the study. (Right panel) The modeling (R2Y) and predictive ability (Q2Y) of OPLS-DA map of samples from feces ((A), 0.998 and 0.646, respectively), urine ((B), 0.952 and 0.108, respectively) and serum ((C), 0.901 and 0.215, respectively). To avoid over-fitting of the model, 999 random permutation tests were performed so as to cross-validate the three components. Their intercept values were −0.071, −0.021, and −0.188 for feces (A), urine (B) and serum (C), respectively, thereby demonstrating the model’s effectiveness; Figure S4: Representative metabolite constituents and proportions obtained through LC-MS metabolomics and the JiaLib^TM^ standard library, which contains more than 1500 endogenous metabolites; Figure S5: Identification of the differential expressing candidate metabolites in feces (A), urine (B) and serum (C) samples in the Test set. (Left panel) The volcano plot (multi-dimensional analysis) revealed the differential expressing metabolites in feces (A), urine (B) and serum (C) samples through the multi-criteria assessment. The green and blue cross shaped buddings present the metabolites within the cutoff value of variable importance in the project (VIP) ≥ 1 and *p*-value < 0.05 and 0.01, respectively. The metabolites in the upper right corner in the image shown upregulation in the fatty liver samples, and metabolites in the upper left corner shown downregulation. (Middle panel) *t* test map (single-dimensional analysis) of the fecal (A), urine (B) and serum (C) samples. The green dots present the metabolites within the cutoff values of *p*-value < 0.05 and the log(fold change) ≥1.2. (Right panel) Venn diagram displays the differential expressing metabolites in the feces (A), urine (B) and serum (C) samples under multi-dimensional and single-dimensional data analysis. Green and blue represent the number of metabolites identified by single- and multi- dimensional analysis, respectively. More detailed information about the intersected common metabolites was listed in Table S5–S7; Figure S6: Diagnostic performance examination of the traditional serum biochemical indicators using ROC (receiver operating characteristic) curve. The line with 45° slope in each panel is the reference boundary line with AUC (area under the curve) of 0.5, meaning the diagnostic method is effective or not. The higher AUC value is, the higher accuracy the diagnostic method is. The area under courve (AUC) values were listed in Table 2; Figure S7: Z-score Heatmap of the finally indentified differentially expressing metabolites in Verification set, a third liver biopsy diagnosed dairy population. Norm represents serum samples from cows with normal liver (*n* = 12), and High represnts serum samples from fatty liver cows in their early postpartum period (*n* = 8); Table S1: Information of biochemical serum indicators of the perinatal dairy cows involved in the Discovery set and Test set in the study; Table S2: Fecal candidate biomarkers identified by single- and multi-dimensional screening in the Discovery set; Table S3: Urine candidate biomarkers identified by single- and multi-dimensional screening in the Discovery set; Table S4: Serum candidate biomarkers identified by single- and multi-dimensional screening in the Discovery set; Table S5: Fecal candidate biomarkers identified by single- and multi-dimensional screening in the Test set; Table S6: Urine candidate biomarkers identified by single- and multi-dimensional screening in the Test set; Table S7: Serum candidate biomarkers identified by single- and multi-dimensional screening in the Test set; Table S8: Significant pathways enriched by deferentially expressing metabolites in serum and feces from dairy cows in the Discovery set and Test set.

## Abbreviations

AFP: alpha-fetoprotein; AST, aspartate aminotransferase; ALB, albumin; ALT, alanine amiotransferase; AUC, area under curve; BHB, β-hydroxybutyric acid; GLU, glucose; HCC, hepatocellular carcinoma; INS insulin; NEFA, non-esterified fatty acids; SUN, serum urea nitrogen; TCHO, total cholesterol; TG, triglyceride; TP, total protein; UA, urea acid; UERA, urea nitrogen; FL, fatty liver; GC–MS, gas chromatography–mass spectrometry; GC–TOFMS, gas chromatography–time-of-flight mass spectrometry; GNMT, glycine N-methyltransferase; KEGG, Kyoto encyclopedia of genes and genomes; NAFLD, non-alcoholic fatty liver disease; NASH, non-alcoholic steatohepatitis; NMR, nuclear magnetic resonance; PCA, principal component analysis; OPLS-DA, orthogonal partial least squares discriminant analysis; ROC, receiver operating characteristic; TCA cycle, tricarboxylic acid cycle; VIP, variable importance in projection.
