# Supplementary material for: Multi-Channel Metabolomics Analysis Identifies Novel Metabolite Biomarkers for the Early Detection of Fatty Liver Disease in Dairy Cows
Source: Cells. 2022 Sep 15;11(18):2883. doi: 10.3390/cells11182883 (PMC9496829; doi:10.3390/cells11182883)
Supplement: Supplementary file 1 [file cells-11-02883-s001.zip › cells-1912118-supplementary.pdf]

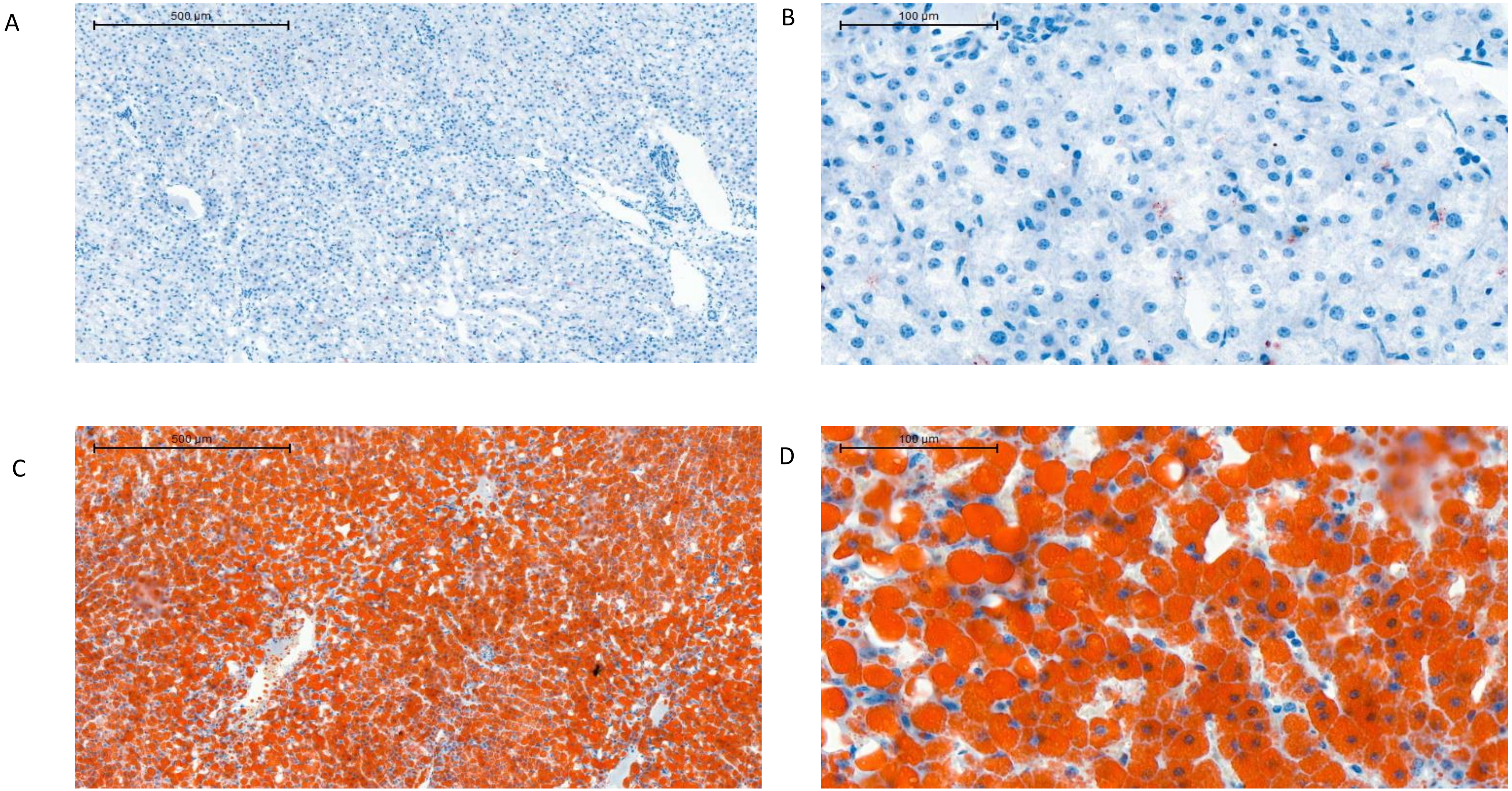

Fig. S1 Oil red O staining revealed the significant lipid accumulation in the fatty liver tissue (C and D), compared with normal control animal (A and B). Red represents stained fat in the cell.

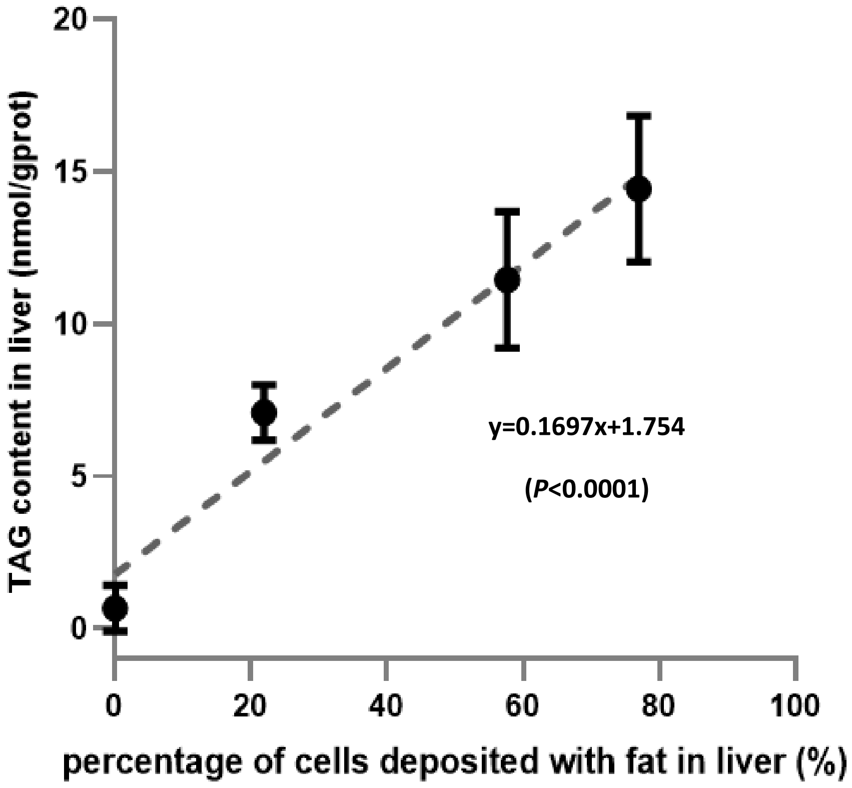

Fig. S2 The TAG content (nmol/g protein) in liver were assessed in representative normal of fatty liver samples, indicating that the values of fatty cell percentage (%) quantitated by liver biopsy in the study were positively correlated with liver TAG content.

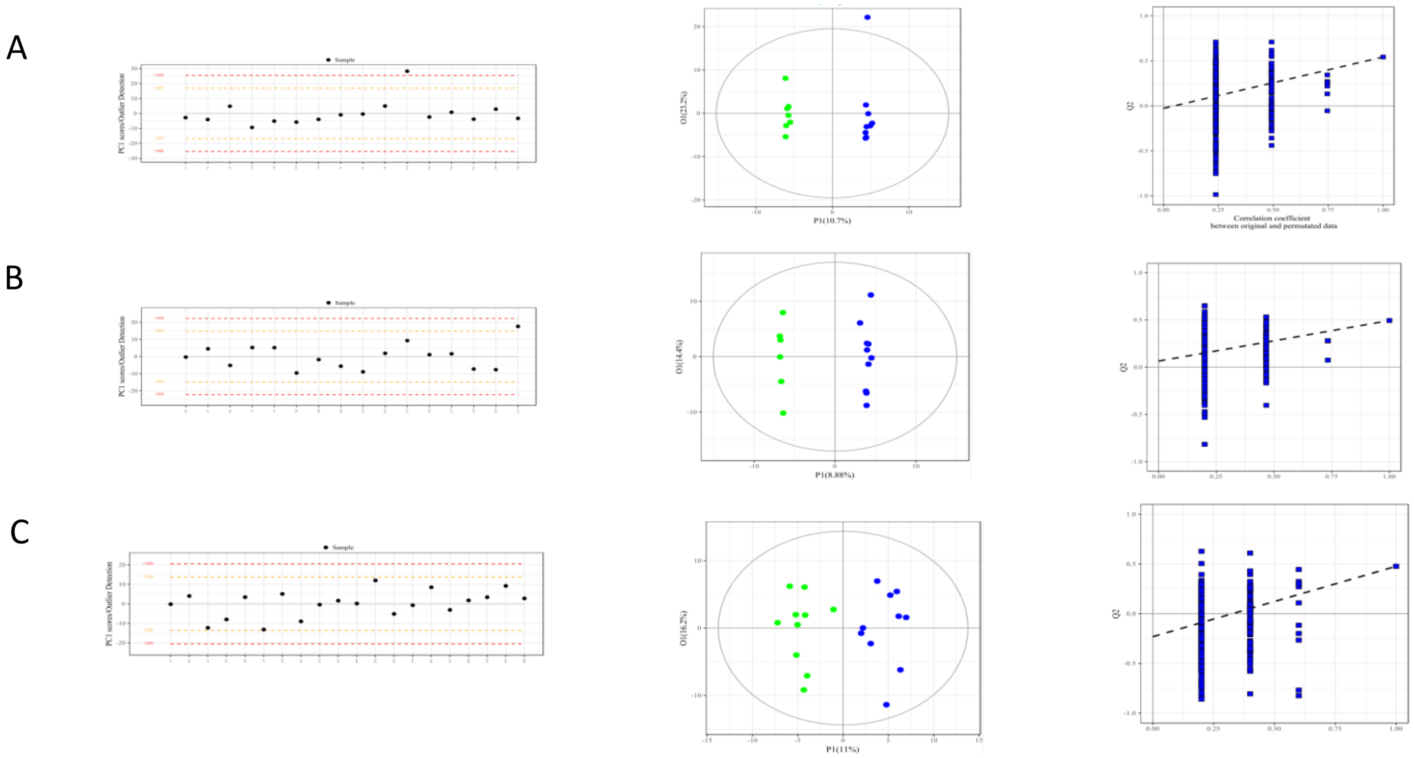

Fig. S3. Quality control and model credibility assessment of samples in the Test set (cows strictly selected by serological detection) from feces (A), urine (B) and serum (C). (Left panel) Multivariate control chart (MCC) shown the metabolite profiles of all individual feces (A), urine (B) and serum (C) samples in the Discovery set. Black dot represents a single sample. Orange and/or red dashed line represents biological quality control range limit. (Middle panel) Orthogonal partial least squares discriminant analysis (OPLS-DA) maps of the samples from feces (A), urine (B) and serum (C), revealing a significant separation of fatty liver group from normal control group, without any overlapping, indicating a successful model construction in the study. (Right panel) The modeling (R2Y) and predictive ability (Q2Y) of OPLS-DA map of samples from feces (A, 0.998 and 0.646, respectively), urine (B, 0.952 and 0.108, respectively) and serum (C, 0.901 and 0.215, respectively). To avoid over-fitting of the model, 999 random permutation tests were performed so as to cross-validate the three components. Their intercept values

were -0.071, -0.021, and -0.188 for feces (A), urine (B) and serum (C), respectively, thereby demonstrating the model's effectiveness.

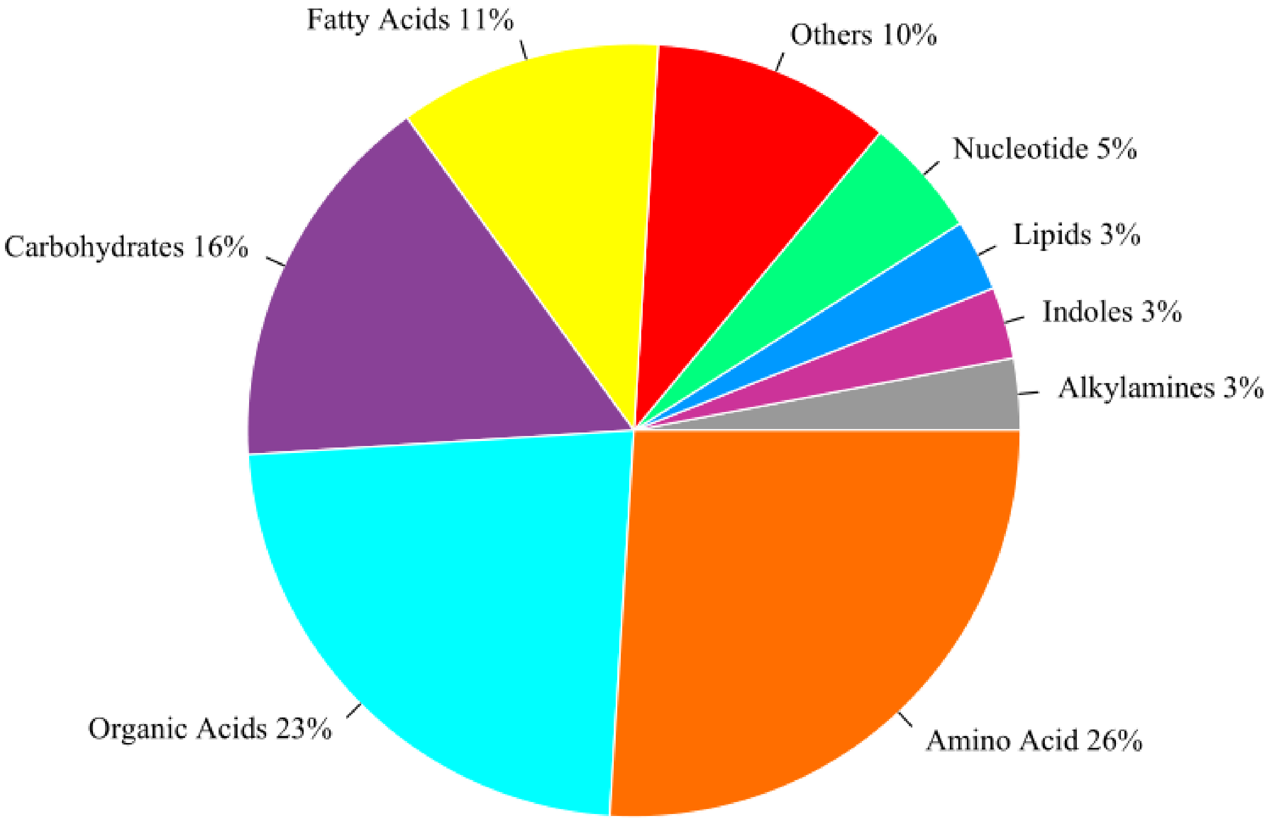

Fig. S4. Representative metabolite constituents and proportions obtained through LC-MS metabolomics and the JiaLibTM standard library, which contains more than 1,500 endogenous metabolites.

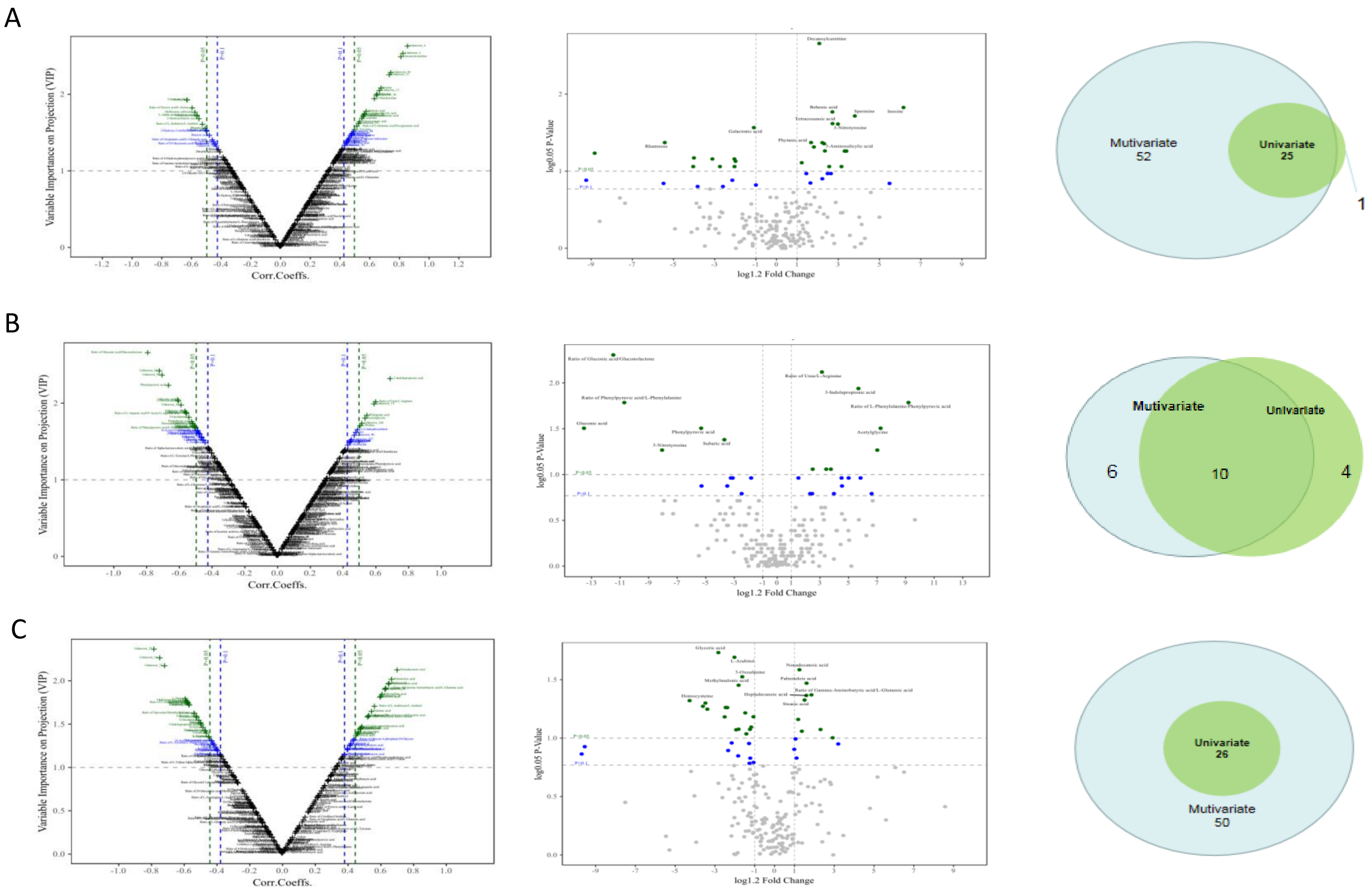

Fig.S5. Identification of the differential expressing candidate metabolites in feces (A), urine (B) and serum (C) samples in the Test set. (Left panel) The volcano plot (multi-dimensional analysis) revealed the differential expressing metabolites in feces (A), urine (B) and serum (C) samples through the multi-criteria assessment. The

green and blue cross shaped buddings present the metabolites within the cutoff value of variable importance in the project (VIP) ≥ 1 and P-value < 0.05 and 0.01, respectively. The metabolites in the upper right corner in the image shown upregulation in the fatty liver samples, and metabolites in the upper left corner shown downregulation. (Middle panel) T test map (single-dimensional analysis) of the fecal (A), urine (B) and serum (C) samples. The green dots present the metabolites within the cutoff values of P-value < 0.05 and the log(fold change) ≥1.2. (Right panel) Venn diagram displays the differential expressing metabolites in the feces (A), urine (B) and serum (C) samples under multi-dimensional and single-dimensional data analysis. Green and blue represent the number of metabolites identified by single- and multi- dimensional analysis, respectively. More detailed information about the intersected common metabolites was listed in Table S5-S7.

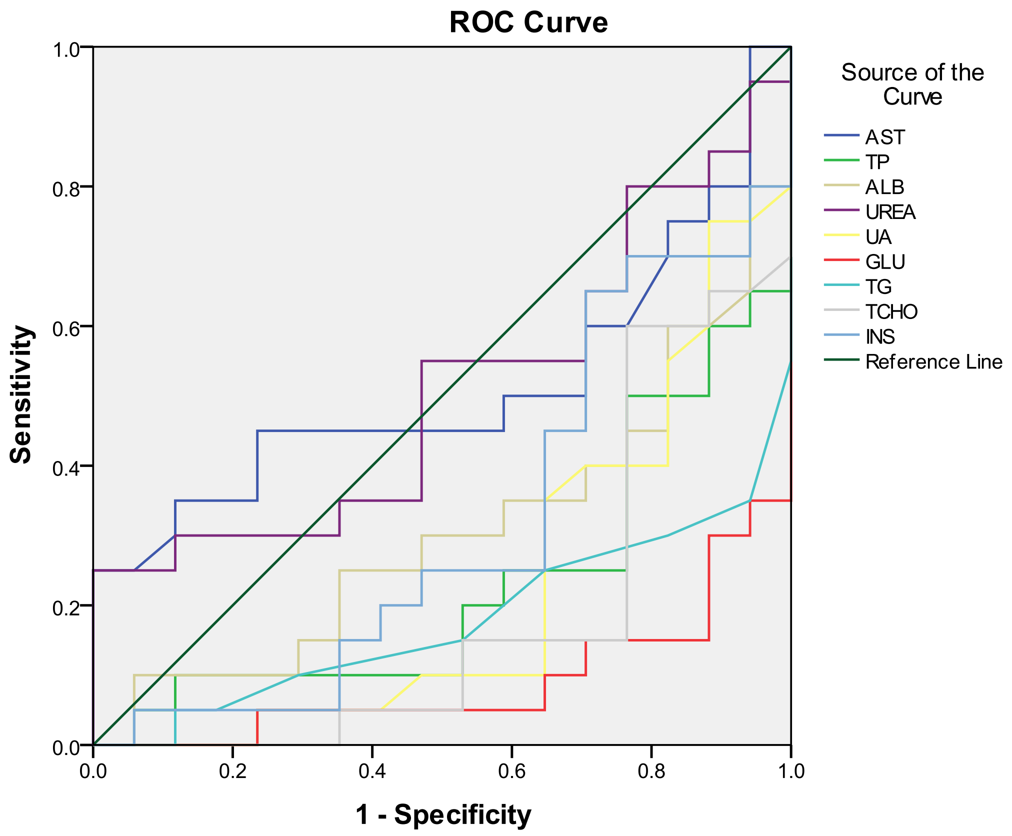

Fig. S6. Diagnostic performance examination of the traditional serum biochemical indicators using ROC (receiver operating characteristic) curve. The line with 45°slope in each panel is the reference boundary line with AUC (area under the curve) of 0.5, meaning the diagnostic method is effective or not. The higher AUC value is, the higher accuracy the diagnostic method is. The area under courve (AUC) values were listed in Table 2.

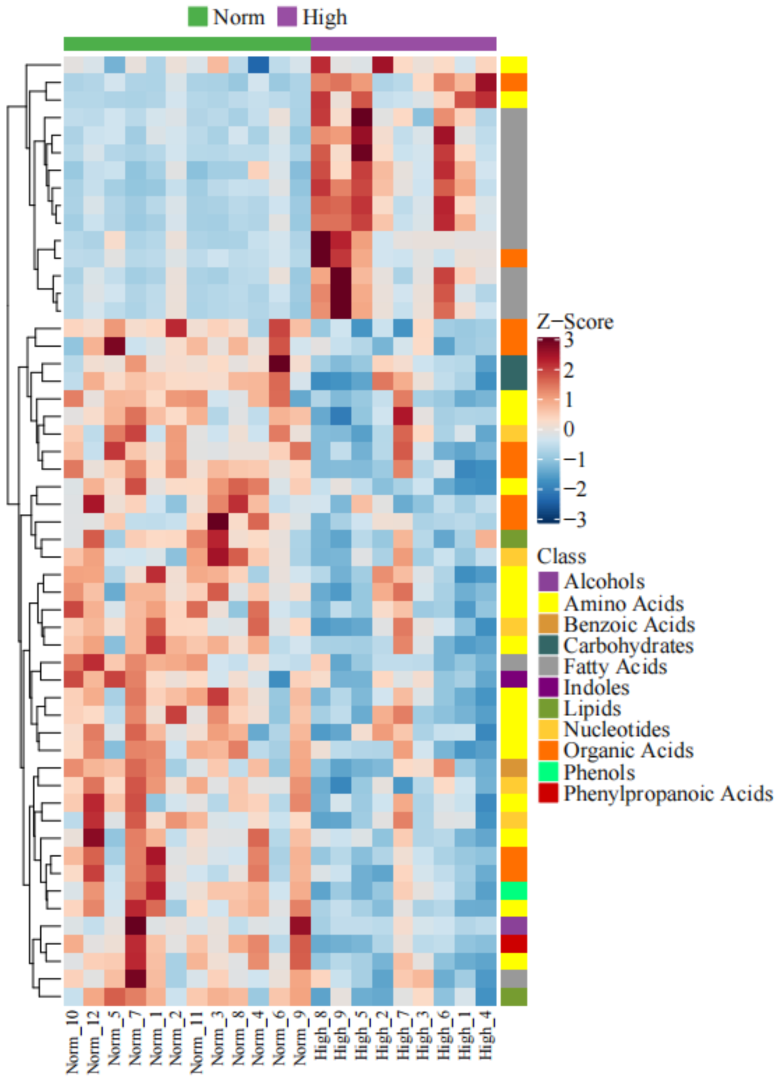
Norm
High
Z−Score
3
2
1
0
−1
−2
−3
Class
Alcohols
Amino Acids
Benzoic Acids
Carbohydrates
Fatty Acids
Indoles
Lipids
Nucleotides
Organic Acids
Phenols
Phenylpropanoic Acids
Norm_10
Norm_12
Norm_5
Norm_7
Norm_1
Norm_2
Norm_11
Norm_3
Norm_8
Norm_4
Norm_6
Norm_9
High_8
High_9
High_5
High_2
High_7
High_3
High_6
High_1
High_4

Fig. S7. Z-score Heatmap of the finally indentified differentially expressing metabolites in Verification set, a third liver biopsy diagnosed dairy population. Norm represents serum samples from cows with normal liver (n=12), and High represnts serum samples from fatty liver cows in their early postpartum period (n=8).

Table S1. Information of biochemical serum indicators of the perinatal dairy cows involved in the Discovery set [1] and Test set [2] in the study

**Serum index [3]**	**Discovery set**			**Test set**		
	**Normal (n=8)**	**FL (n=10)**	***P* value**	**Suspected Normal (n=10)**	**Suspected FL (n=10)**	***P* value**
AST (IU/L)	89.00±23.88	173.00±110.68	0.061	88.86±20.36	171.55±40.21	0.052
TP (g/L)	71.58±11.54	66.32±10.48	0.32	67.01±7.08	49.83±6.84	2.29E-04
NEFA (mmol/L)	1.39±0.85	2.93±2.06	0.066	1.41±0.61	4.25±1.83	0.0016
OHB(mmol/L）	0.78±0.88	1.58±2.10	0.67	0.47±1.03	1.83±0.92	0.27
GLU(mmol/L)	3.08±0.55	2.12±0.81	0.011	3.17±0.44	2.23±0.22	7.47E-05
INS (pmol/ml)	0.36±0.34	0.24±0.30	0.43	0.31±0.34	0.25±0.13	0.16
TG (g/L)	0.13±0.029	0.11±0.033	0.23	0.13±0.03	0.084±0.016	0.0011
TCHO (g/L)	2.08±0.55	1.62±0.26	0.032	2.23±0.58	1.45±0.29	0.0036
ALB (g/L)	29.16±3.51	29.12±3.38	0.98	30.51±3.17	23.90±3.27	0.0012
UREA (μmol/L)	3.34±0.80	4.05±2.27	0.41	4.22±1.00	3.95±1.12	0.64
UA (mol/L)	39.13±7.72	33.5±10.36	0.22	43.85±16.89	27.44±8.23	0.022
Parity No.	1.13±0.35	1.50±0.71	0.19	1.29±0.49	1.22±0.44	0.79
Day in milk (day)	7±2	7±2	1.00	7±2	7±2	1.00
Body weight (kg)	650±53	668±45	0.34	651±39	658±48	0.62

[1] Discovery set, samples from cows diagnosed by liver biopsy.

[2] Test set, samples from cows diagnosed by serology.

[3] AST, aspartate aminotransferase; TP, total protein; NEFA, nonesterified fatty acid; OHB, β-Hydroxybutyrate; GLU, glucose; INS, insulin; TG, triglyceride; TCHO, total cholesterol; ALB,

albumin;UREA, urea nitrogen;UA, urea acid.

Table. S2 Fecal candidate biomarkers identified by single- and multi-dimensional screening in the Discovery set [1]

Class	Biomarker Name	*P* value	FC [2]	HMDB ID [3]	KEGG ID [4]	Associated Pathway
/	3-hydroxypyridine	4.50E-04	0.403	/	/	/
Organic Acids	3-Pyridylacetic acid	5.20E-04	0.534	HMDB01538	/	/
	Petroselinic acid	2.70E-02	0.664	HMDB02080	C08363	/
	Fumaric acid	2.00E-02	0.745	HMDB00134	C00122	Arginine and Proline Metabolism; Aspartate Metabolism; Citric Acid Cycle; Mitochondrial Electron Transport Chain; Phenylalanine and Tyrosine Metabolism; Tyrosine Metabolism; Urea Cycle
Vitamin	Alpha-Tocopherol	1.40E-03	0.545	HMDB01893	C02477	/
Amino Acid	3-Aminoisobutanoic acid	4.40E-03	0.47	HMDB03911	C05145	Pyrimidine Metabolism
	Ketoleucine	4.80E-03	1.256	HMDB00695	C00233	Valine, Leucine and Isoleucine Degradation
	L-Alpha-aminobutyric acid	8.50E-03	0.534	HMDB00452	C02356	/
	5-Aminopentanoic acid	2.70E-02	0.382	HMDB03355	C00431	/
	Glycine	2.70E-02	0.313	HMDB00123	C00037	Alanine Metabolism,Ammonia Recycling; Bile Acid Biosynthesis; Carnitine Synthesis; Glutathione Metabolism; Glycine and Serine Metabolism, Methionine Metabolism; Porphyrin Metabolism
	Urea	3.20E-02	0.154	HMDB00294	C00086	Arginine and Proline Metabolism; Urea Cycle
Amino Acid	L-Glutamine	3.40E-02	0.617	HMDB00641	C00064	Amino Sugar Metabolism,Ammonia Recycling; Glutamate Metabolism; Phenylacetate Metabolism; Purine Metabolism; Pyrimidine Metabolism; Transcription/Translation; Urea

						Cycle
	Acetylglycine	3.70E-02	0.789	HMDB00532	/	/
	Ratio of L-Asparagine/L-Aspartic acid	4.10E-02	0.547	HMDB00168/HMDB00191	C00152/C00049	Ammonia Recycling; Aspartate Metabolism,Transcription/Translation; Ammonia Recycling; Arginine and Proline Metabolism; Aspartate Metabolism; Beta-Alanine Metabolism; Malate-Aspartate Shuttle; Transcription/Translation; Urea Cycle
Phenols	m-Cresol	8.50E-03	0.173	HMDB02048	C01467	/
Alcohols	Myoinositol	8.70E-03	0.652	HMDB00211	C00137	Galactose Metabolism; Inositol Metabolism; Inositol Phosphate Metabolism; Phosphatidylinositol Phosphate Metabolism
Aromatic aldehydes	3,4-Dihydro-2H-1-benzopyran-2-one	1.20E-02	0.75	HMDB36626	C02274	/
	Glyceraldehyde	1.80E-02	0.5	HMDB01051	C02154	Glycerolipid Metabolism
Carbohydrates	Glucose 1-phosphate	2.70E-02	0.757	HMDB01586	C00103	Galactose Metabolism; Gluconeogenesis, Glycolysis, Lactose Synthesis; Nucleotide Sugars Metabolism; Starch and Sucrose Metabolism
	D-Glucuronic acid	3.70E-02	0.471	HMDB00127	C00191	Inositol Metabolism; Starch and Sucrose Metabolism
Fatty Acids	Arachidonic acid	2.90E-02	0.442	HMDB01043	C00219	Alpha Linolenic Acid and Linoleic Acid Metabolism; Arachidonic Acid Metabolism
	Behenic acid	4.10E-02	1.203	HMDB00944	C08281	/

[1] In the Discovery set (samples from cows diagnosed by liver biopsy), the feces metabolites with significant difference in the normal and fatty liver dairy cows were identified through VIP multi-dimensional test (VIP > 1) and then single-dimensional *t* test ($P < 0.05$).

[2] FC, fold change, indicating the relative differential expression level of the metabolite in fatty liver and normal dairy cows.

[3] HMDB ID: ID number in Human Metabolome Database that the metabolite corresponds to.

[4] KEGG ID: ID number of the biological information database that the metabolic pathway corresponds to.

Table S3. Urine candidate biomarkers identified by single- and multi-dimensional screening in the Discovery set [1]

Class	Biomarker Name	*P* value	FC [2]	HMDB ID [3]	KEGG ID [4]	Associated Pathway
Amino Acid	Salicyluric acid	7.80E-04	0.439	HMDB00840	C07588	/
	Alpha-ketoisovaleric acid	7.80E-03	1.95	HMDB00019	C00141	Valine, Leucine and Isoleucine Degradation
Amino Acid	Ratio of Alpha-ketoisovaleric acid/L-Valine	7.80E-03	2.776	HMDB00019/HMDB00883	C00141/C00183	Valine, Leucine and Isoleucine Degradation; Propanoate Metabolism,Transcription/Translation, Valine, Leucine and Isoleucine Degradation
	Ratio of L-Valine/Alpha-ketoisovaleric acid	7.80E-03	0.36	HMDB00883/HMDB00019	C00183/C00141	Valine, Leucine and Isoleucine Degradation; Propanoate Metabolism,Transcription/Translation, Valine, Leucine and Isoleucine Degradation
	3-Nitrotyrosine	3.10E-02	0.256	HMDB01904	/	/
Alkylamines	Spermidine	1.90E-02	0.351	HMDB01257	C00315	Methionine Metabolism,Spermidine and Spermine Biosynthesis
Fatty Acids	Linoleic acid	4.00E-02	1.452	HMDB00673	C01595	Alpha Linolenic Acid and Linoleic Acid Metabolism

[1] In the Discovery set (samples from cows diagnosed by liver biopsy), the urine metabolites with significant difference in the normal and fatty liver dairy cows were identified through VIP multi-dimensional test (VIP > 1) and then single-dimensional t test ($P < 0.05$).

[2] FC, fold change, indicating the relative differential expression level of the metabolite in fatty liver and normal dairy cows.

[3] HMDB ID: ID number in Human Metabolome Database that the metabolite corresponds to.

[4] KEGG ID: ID number of the biological information database that the metabolic pathway corresponds to.

Table S4. Serum candidate biomarkers identified by single- and multi-dimensional screening in the Discovery set[1].

Class	Name	FC [2]	*P* value	HMDB ID [3]	KEGG ID [4]	Associated Pathway
Amino Acid	L-Asparagine	0.58	8.70E-04	HMDB00168	C00152	Ammonia Recycling; Aspartate Metabolism,Transcription/Translation
	L-Glutamine	0.514	8.70E-04	HMDB00641	C00064	Amino Sugar Metabolism; Ammonia Recycling; Glutamate Metabolism; Phenylacetate Metabolism; Purine Metabolism; Pyrimidine Metabolism,Transcription/Translation; Urea Cycle
	2-Hydroxybutyric acid	2.016	4.40E-03	HMDB00008	C05984	/
	L-Serine	0.579	2.70E-02	HMDB00187	C00065	Ammonia Recycling; Glycine and Serine Metabolism; Homocysteine Degradation; Methionine Metabolism; Sphingolipid Metabolism
	L-Histidine	0.804	4.30E-02	HMDB00177	C00135	Ammonia Recycling; Histidine Metabolism; Transcription/Translation
	Ratio of L-Tyrosine / L-Phenylalanine	0.685	4.30E-02	HMDB00158/HMDB00159	C00082/C00079	Catecholamine Biosynthesis; Phenylalanine and Tyrosine Metabolism; Transcription/Translation; Tyrosine Metabolism; Phenylalanine and Tyrosine Metabolism; Transcription/Translation
Organic Acids	4-Hydroxybenzoic acid	1.292	4.40E-03	HMDB00500	C00156	Ubiquinone Biosynthesis
/	1-Octanoyl-rac-glycerol	0.427	8.50E-03	/	/	/
Carbohydrates	D-Glucuronic acid	0.531	1.20E-02	HMDB00127	C00191	Inositol Metabolism; Starch and Sucrose Metabolism

	Mannitol	0.597	1.60E-02	HMDB00765	C00392	/
	Sucrose	2.11	2.10E-02	HMDB00258	C00089	Galactose Metabolism; Starch and Sucrose Metabolism
Fatty Acids	Palmitoleic acid	2.191	1.20E-02	HMDB03229	C08362	/
	Palmitic acid	1.989	2.10E-02	HMDB00220	C00249	Fatty Acid Biosynthesis; Fatty Acid Elongation In Mitochondria; Fatty acid Metabolism; Glycerolipid Metabolism
	Nonadecanoic acid	1.678	2.70E-02	HMDB00772	C16535	/
Fatty Acids	Stearic acid	1.819	2.70E-02	HMDB00827	C01530	Mitochondrial Beta-Oxidation of Long Chain Saturated Fatty Acids; Plasmalogen Synthesis
	Arachidic acid	1.394	3.40E-02	HMDB02212	C06425	/
	Heptadecanoic acid	2.272	3.40E-02	HMDB02259	/	/
Lipids	Cholesterol	0.828	1.60E-02	HMDB00067	C00187	Bile Acid Biosynthesis; Steroid Biosynthesis; Steroidogenesis
Esters	Cyclohexaneundecanoic acid	2.07	2.10E-02	HMDB30997	C12100	/
Indoles	Serotonin	0.548	2.10E-02	HMDB00259	C00780	Tryptophan Metabolism
	3-Hydroxybutyric acid	1.682	2.60E-02	HMDB00357	C01089	Fatty Acid Biosynthesis
	2-Hydroxy-3-methylbutyric acid	1.853	2.70E-02	HMDB00407	/	/
Organic Acids	Petroselinic acid	2.831	2.70E-02	HMDB02080	C08363	/
	Taurine	0.562	4.30E-02	HMDB00251	C00245	Bile Acid Biosynthesis,Taurine and Hypotaurine Metabolism

[1] In the Discovery set (samples from cows diagnosed by liver biopsy), the serum metabolites with significant difference in the normal and fatty liver dairy cows were identified through VIP multi-dimensional test (VIP > 1) and then single-dimensional t test ($P < 0.05$).
[2] FC, fold change, indicating the relative differential expression level of the metabolite in fatty liver and normal dairy cows.
[3] HMDB ID: ID number in Human Metabolome Database that the metabolite corresponds to.
[4] KEGG ID: ID number of the biological information database that the metabolic pathway corresponds to.

Table S5. Fecal candidate biomarkers identified by single- and multi-dimensional screening in the Test set [1].

Class	Name	FC [2]	*P* value	HMDB ID [3]	KEGG ID [4]	Associated Pathway
Organic Acids	3-Aminosalicylic acid	1.5	1.70E-02	HMDB01972	/	/
Fatty Acids	Arachidic acid	1.535	2.30E-02	HMDB02212	C06425	/
	Behenic acid	1.64	5.00E-03	HMDB00944	C08281	/
	Tetracosanoic acid	1.641	7.90E-03	HMDB02003	C08320	Beta Oxidation of Very Long Chain Fatty Acids
Lipids	Decanoylcarnitine	1.46	3.50E-04	HMDB00651	/	/
Carbohydrates	Erythrose	0.481	3.00E-02	HMDB02649	C01796	/
	Galactonic acid	0.818	9.20E-03	HMDB00565	C00880	/
	Rhamnose	0.372	1.60E-02	HMDB00849	C00507	/
Nucleotide	Inosine	3.077	4.20E-03	HMDB00195	C00294	Purine Metabolism
Amino Acid	2-Hydroxybutyric acid	0.606	4.20E-02	HMDB00008	C05984	/
	3-Nitrotyrosine	1.722	8.00E-03	HMDB01904	/	/
	Creatinine	1.392	2.00E-02	HMDB00562	C00791	/
	L-Alpha-aminobutyric acid	0.696	3.40E-02	HMDB00452	C02356	/
	L-Lysine	1.855	2.30E-02	HMDB00182	C00047	Biotin Metabolism; Carnitine Synthesis; Lysine Degradation; Transcription/Translation
	L-Tyrosine	1.827	2.30E-02	HMDB00158	C00082	Catecholamine Biosynthesis; Phenylalanine and Tyrosine Metabolism; Transcription/Translation; Tyrosine Metabolism
	Methionine sulfoxide	0.2	2.50E-02	HMDB02005	/	Methionine Metabolism
	Ratio of L-Glutamic acid/Pyroglutamic acid	1.777	4.20E-02	HMDB00148/ HMDB00267	C00025/C01879	Alanine Metabolism; Amino Sugar Metabolism; Ammonia Recycling; Arginine and Proline Metabolism; Cysteine Metabolism; Folate Metabolism; Glucose-Alanine Cycle;

						Glutamate Metabolism; Glutathione Metabolism; Glycine and Serine Metabolism; Histidine Metabolism; Malate-Aspartate Shuttle; Transcription/Translation; Urea Cycle; Glutathione Metabolism
	Ratio of Pyruvic acid/L-Serine	0.567	3.10E-02	HMDB00243/HMDB00187	C00022/C00065	Ammonia Recycling; Glycine and Serine Metabolism; Homocysteine Degradation; Methionine Metabolism; Sphingolipid Metabolism; Alanine Metabolism; Amino Sugar Metabolism; Ammonia Recycling; Citric Acid Cycle; Cysteine Metabolism; Gluconeogenesis, Glucose-Alanine Cycle; Glycine and Serine Metabolism; Glycolysis; Pyruvaldehyde Degradation; Pyruvate Metabolism; Transfer of Acetyl Groups into Mitochondria; Urea Cycle
Alkylamines	Spermine	2	5.80E-03	HMDB01256	C00750	Spermidine and Spermine Biosynthesis

[1] In the Test set (samples from cows diagnosed by serology), the feces metabolites with significant difference in the normal and fatty liver dairy cows were identified through VIP multi-dimensional test (VIP > 1) and then single-dimensional t test ($P < 0.05$).

[2] FC, fold change, indicating the relative differential expression level of the metabolite in fatty liver and normal dairy cows.

[3] HMDB ID: ID number in Human Metabolome Database that the metabolite corresponds to.

[4] KEGG ID: ID number of the biological information database that the metabolic pathway corresponds to.

Table. S6 Urine candidate biomarkers identified by single- and multi-dimensional screening in the Test set [1].

Class	Name	FC [2]	*P* value	HMDB ID [3]	KEGG ID [4]	Associated Pathway
Indoles	3-Indolepropionic acid	2.825	3.00E-03	HMDB02302	/	/
Amino Acid	3-Nitrotyrosine	0.23	2.20E-02	HMDB01904	/	/
	Acetylglycine	3.748	1.10E-02	HMDB00532	/	/
	Ratio of Urea/L-Arginine	1.772	1.70E-03	HMDB00294/HMDB00517	C00086/C00062	Arginine and Proline Metabolism; Urea Cycle; Arginine and Proline Metabolism; Glycine and Serine Metabolism; Transcription/Translation; Urea Cycle
Carbohydrates	Gluconic acid	0.085	1.10E-02	HMDB00625	C00257	/
	Ratio of Gluconic acid / Gluconolactone	0.123	1.00E-03	HMDB00625/HMDB00150	C00257/C00198	Pentose Phosphate Pathway
Fatty Acids	Pelargonic acid	3.593	2.20E-02	HMDB00847	C01601	/
Organic Acids	Phenylpyruvic acid	0.379	1.10E-02	HMDB00205	C00166	Phenylalanine and Tyrosine Metabolism
	Suberic acid	0.51	1.60E-02	HMDB00893	C08278	/

[1] In the Test set (samples from cows diagnosed by serology), the urine metabolites with significant difference in the normal and fatty liver dairy cows were identified through VIP multi-dimensional test (VIP > 1) and then single-dimensional t test ($P < 0.05$).

[2] FC, fold change, indicating the relative differential expression level of the metabolite in fatty liver and normal dairy cows.

[3] HMDB ID: ID number in Human Metabolome Database that the metabolite corresponds to.

[4] KEGG ID: ID number of the biological information database that the metabolic pathway corresponds to.

Table S7. Serum candidate biomarkers identified by single- and multi-dimensional screening in the Test set [1]

Class	Name	FC [2]	*P* value	HMDB ID [3]	KEGG ID [4]	Associated Pathway
Carbohydrates	D-Galactose	0.764	2.60E-02	HMDB00143	C00984	Galactose Metabolism; Lactose Degradation; Nucleotide Sugars Metabolism
	D-Xylose	0.824	2.90E-02	HMDB00098	C00181	/
	L-Arabitol	0.692	6.30E-03	HMDB01851	C00532	/
	Ratio of L-Arabinose/L-Arabitol	1.241	3.10E-02	HMDB00646/HMDB01851	C00259/C00532	/
	Ribitol	0.703	4.00E-02	HMDB00508	C00474	/
Organic Acids	Glyceric acid	0.597	5.60E-03	HMDB00139	C00258	Glycerolipid Metabolism; Glycine and Serine Metabolism
	Hippuric acid	0.53	2.00E-02	HMDB00714	C01586	/
	Methylmalonic acid	0.719	1.30E-02	HMDB00202	C02170	Propanoate Metabolism; Valine, Leucine and Isoleucine Degradation
	Petroselinic acid	1.524	4.00E-02	HMDB02080	C08363	/
Amino Acid	Homocysteine	0.459	1.90E-02	HMDB00742	NA	Betaine Metabolism; Catecholamine Biosynthesis; Glycine and Serine Metabolism; Homocysteine Degradation; Methionine Metabolism
	3-Oxoalanine	0.743	1.00E-02	HMDB11602	NA	/
	L-Asparagine	0.648	2.30E-02	HMDB00168	C00152	Ammonia Recycling; Aspartate Metabolism; Transcription/Translation
	L-Cysteine	0.798	4.00E-02	HMDB00574	NA	Cysteine Metabolism; Glutathione Metabolism; Glycine and Serine Metabolism; Methionine Metabolism; Pantothenate and CoA Biosynthesis; Taurine and Hypotaurine Metabolism;

						Transcription/Translation
	L-Cystine	0.638	2.30E-02	HMDB00192	C00491	/
	L-Serine	0.518	2.20E-02	HMDB00187	C00065	Ammonia Recycling; Glycine and Serine Metabolism; Homocysteine Degradation; Methionine Metabolism; Sphingolipid Metabolism
	Ratio of Gamma-Aminobutyric acid/L-Glutamic acid	1.401	1.70E-02	HMDB00112/HMDB00148	C00334/C00025	Glutamate Metabolism; Alanine Metabolism; Amino Sugar Metabolism; Ammonia Recycling; Arginine and Proline Metabolism; Cysteine Metabolism; Folate Metabolism; Glucose-Alanine Cycle; Glutamate Metabolism; Glutathione Metabolism; Glycine and Serine Metabolism; Histidine Metabolism; Malate-Aspartate Shuttle; Transcription/Translation; Urea Cycle
Fatty Acids	Myristic acid	1.284	4.20E-02	HMDB00806	C06424	Fatty Acid Biosynthesis
	Nonadecanoic acid	1.256	8.70E-03	HMDB00772	C16535	/
	Palmitoleic acid	1.34	1.20E-02	HMDB03229	C08362	/
	Heptadecanoic acid	1.338	1.70E-02	HMDB02259	NA	/
	Stearic acid	1.316	1.90E-02	HMDB00827	C01530	Mitochondrial Beta-Oxidation of Long Chain Saturated Fatty Acids; Plasmalogen Synthesis
Nucleotide	Uridine	0.54	2.40E-02	HMDB00296	C00299	Pyrimidine Metabolism

[1] In the Test set (samples from cows diagnosed by serology), the serum metabolites with significant difference in the normal and fatty liver dairy cows were identified through VIP multi-dimensional test (VIP > 1) and then unit t test ($P < 0.05$).

[2] FC, fold change, indicating the relative differential expression level of the metabolite in fatty liver and normal dairy cows.

[3] HMDB ID: ID number in Human Metabolome Database that the metabolite corresponds to.

[4] KEGG ID: ID number of the biological information database that the metabolic pathway corresponds to.

Table S8. Significant pathways enriched by deferentially expressing metabolites in serum and feces from dairy cows in the Discovery set [1] and Test set [2].

Metabolite sources	Pathway Name	*P*-hyper	Up-regulated metabolites	Down-regulated metabolites
Serum	Arginine biosynthesis	<0.01	Fumaric acid	L-Arginine; L-Aspartic acid; L-Glutamine
	Alanine, aspartate and glutamate metabolism	<0.01	Fumaric acid	L-Alanine; L-Asparagine; L-Aspartic acid; L-Gluta mine
	Arginine and proline metabolism	<0.05	Spermidine	4-Hydroxyproline; Creatine; L-Arginine; L-Proline
	Biosynthesis of unsaturated fatty acids	<0.05	Arachidic acid; Oleic acid; Palmitic acid; Stearic acid	
	Histidine metabolism	<0.05		1-Methylhistidine; L-Aspartic acid; L-Histidine
	Cysteine and methionine metabolism	<0.05		Homocysteine; L-Cysteine; L-Methionine; L-Serine
	Pantothenate and CoA biosynthesis	<0.05		L-Aspartic acid; L-Cysteine; Pantothenic acid
	Glycine, serine and threonine metabolism	<0.05		Creatine; L-Cysteine; L-Serine; L-Threonine
	Taurine and hypotaurine metabolism	<0.05		L-Cysteine; Taurine
	beta-Alanine metabolism	<0.05	Spermidine	L-Aspartic acid; L-Histidine
Feces	Pentose and glucuronate interconversions	<0.01		D-Glucuronic acid; D-Xylose; Glucose 1-phosphate; L-Arabitol

[1] Discovery set, samples from cows diagnosed by liver biopsy.

[2] Test set, samples from cows diagnosed by serological detection.
